# Supplementary material for: CDK9 and PP2A regulate RNA polymerase II transcription termination and coupled RNA maturation
Source: EMBO Rep. 2022 Aug 18;23(10):e54520. doi: 10.15252/embr.202154520 (PMC9535751; doi:10.15252/embr.202154520)
Supplement: Supplementary file 1 — Appendix S1 [file EMBR-23-e54520-s003.pdf]

CDK9 and PP2A regulate RNA polymerase II transcription termination and coupled RNA maturation.

Michael Tellier[1*], Justyna Zaborowska[1], Jonathan Neve[2], Takayuki Nojima[3], Svenja Hester[2], Marjorie Fournier[2], Andre Furger[2], Shona Murphy[1 *]

[1] Sir William Dunn School of Pathology, University of Oxford, South Parks Road, Oxford OX1 3RE, UK.

[2] Department of Biochemistry, University of Oxford, South Parks Road, Oxford OX1 3QU, UK.

[3] Medical Institute of Bioregulation, Kyushu University, 3-1-1 Maidashi, Higashi-ku, Fukuoka 812-8582, Japan

*For correspondence: michael.tellier@path.ox.ac.uk (MT); shona.murphy@path.ox.ac.uk (SM)

Table of contents

Appendix Figure S1. CDK9 inhibition abrogates polyadenylation……………………………………..2

Appendix Figure S2. Inhibition of a CDK9 analog sensitive (as) cell line produces similar results to the current CDK9 inhibitors…………………………………………………………………………....4

Appendix Figure S3. CDK9 phosphorylates several transcription and splicing factors *in vivo*…….6

Appendix Figure S4. PP1 and PP2A regulates transcription of protein-coding genes……………...8

Appendix Figure S5. CDK9 and PP2A regulate mRNA cleavage and polyadenylation…………...10

Appendix Table S1: List of antibodies used in mNET-seq, ChIP, co-immunoprecipitation, and western blots 12

Appendix Table 2: List of primers used for ChIP-qPCR and qRT PCR ………………………..13

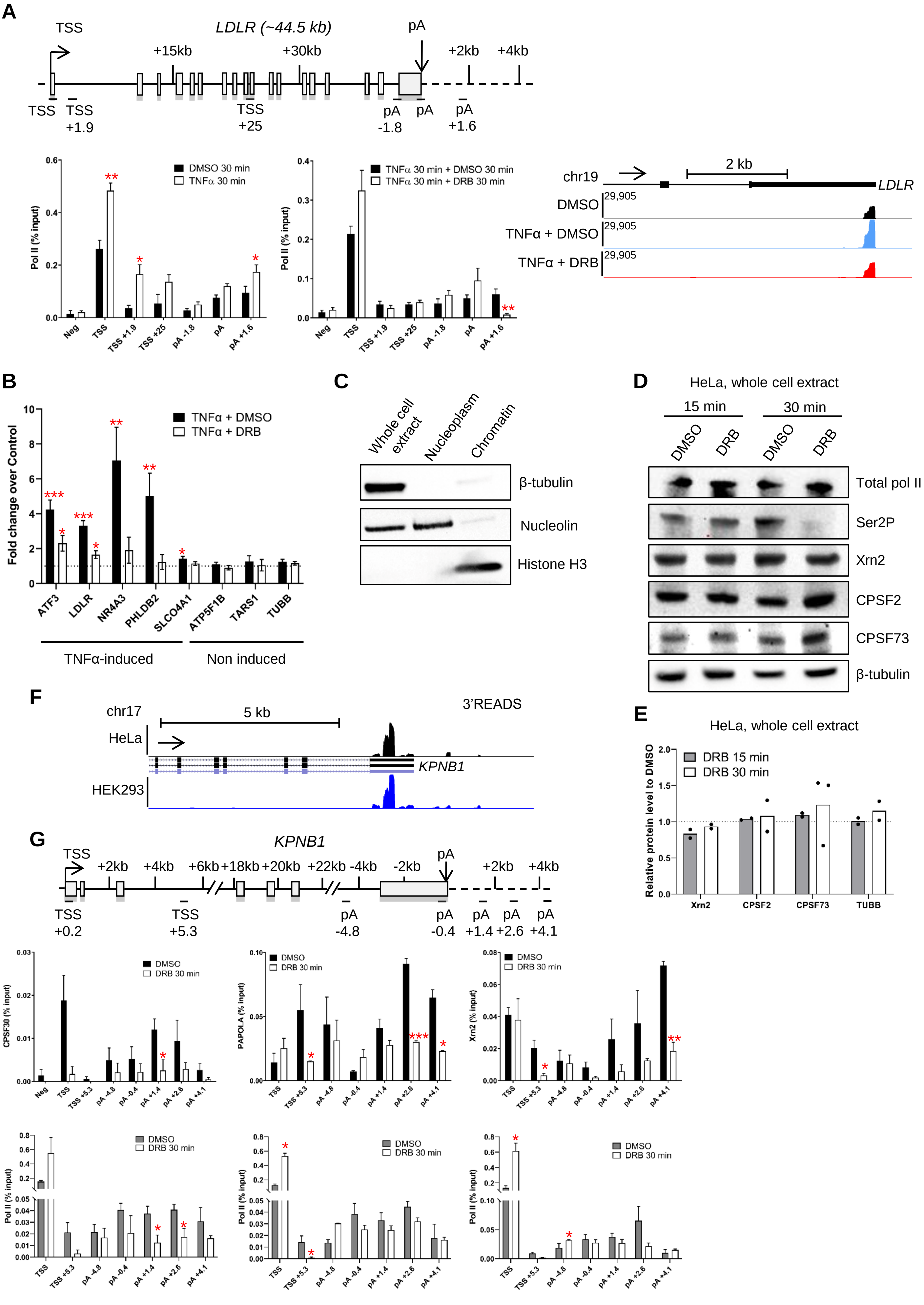

Appendix Figure S1

Appendix Figure S1. CDK9 inhibition abrogates polyadenylation.

A. Pol II ChIP-qPCR on the TNFα-induced gene LDLR and screenshot of the genome browser track of the 3'READS experiments for this gene. n=3 biological replicates, mean ± SEM, p-value: * $p < 0.05$, ** $p < 0.01$. Statistical test: two-tailed unpaired t test. **B.** qRT-PCR of nuclear polyadenylated mRNAs of several TNFα-induced or non-induced genes with a 30 minutes DMSO or a DRB (100 μM here and in all other figures unless stated otherwise) treatment. n=5 biological replicates, mean ± SEM, p-value: * $p < 0.05$, ** $p < 0.01$, *** $p < 0.001$. Statistical test: two-tailed unpaired t test. **C.** Western blot of β-tubulin, Nucleolin, and Histone H3 on whole cell extract, nucleoplasm, and chromatin fractions. **D**. Western blot of total pol II, Ser2P, Xrn2, CPSF2, CPSF73, and β-tubulin as a loading control, on whole cell extract. **E.** Quantification of the western blots shown in D. n=2 biological replicates except for CPSF73 DRB 30 min where n=3 biological replicates, mean ± SEM. **F.** Screenshot of the genome browser track of the 3'READS experiments for *KPNB1* showing the usage of two poly(A) sites in HEK293 and HeLa cell lines. **G.** ChIP-qPCR of total pol II, CPSF73, PAPOLA, and Xrn2 on *KPNB1*. The CPA factor / total pol II ratios shown in Figure 1F were calculated based on the pol II profile located below each CPA factor ChIP-qPCR profile. n=3 biological replicates, mean ± SEM, p-value: * $p < 0.05$, ** $p < 0.01$, *** $p < 0.001$. Statistical test: two-tailed unpaired t test.

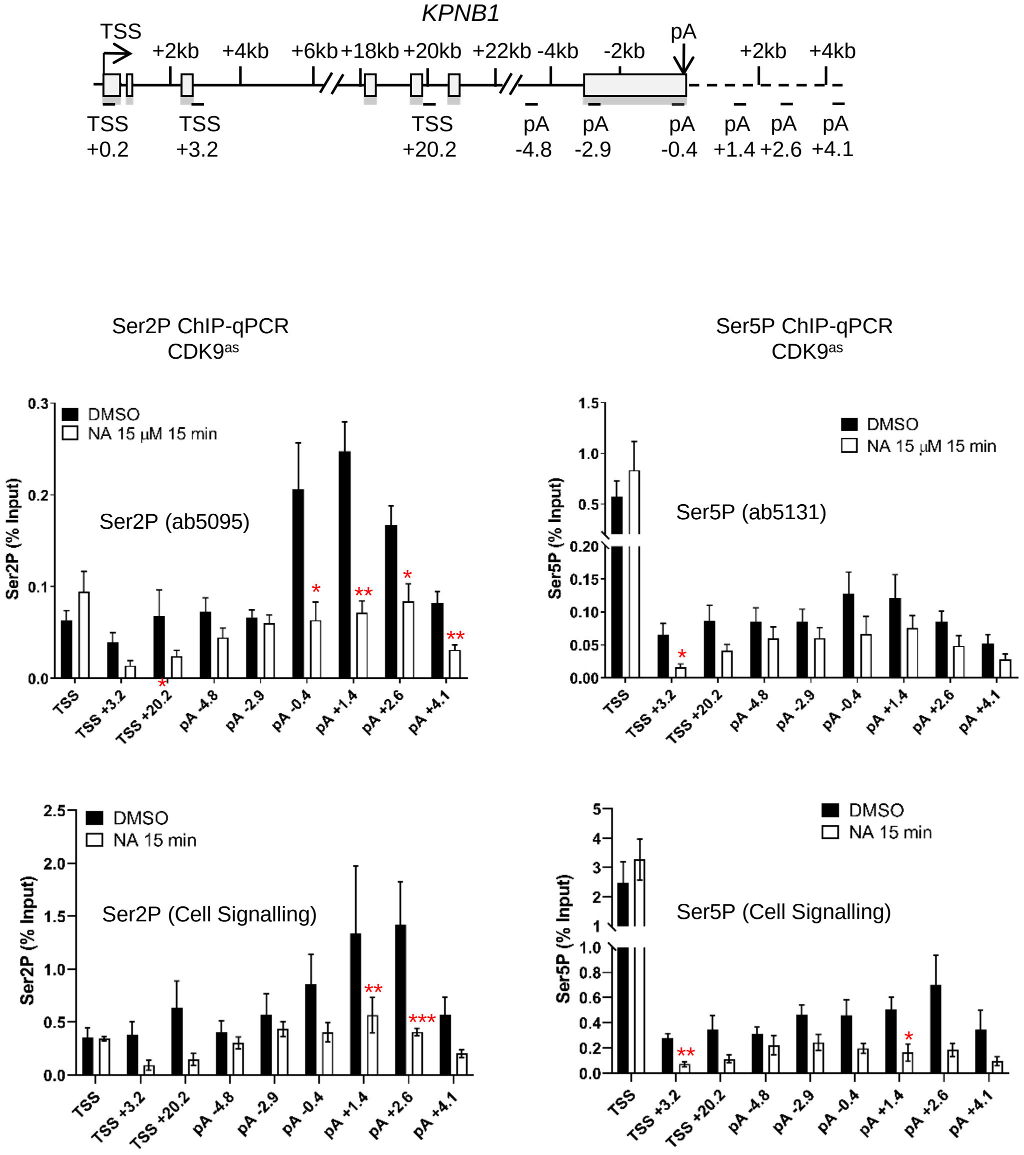

Appendix Figure S2

Appendix Figure S2. Inhibition of analog sensitive CDK9 (as) produces similar results to the current CDK9 inhibitors.

ChIP-qPCR of Ser2P (Abcam or Cell Signaling), and Ser5P (Abcam or Cell Signaling) in CDK9as cells treated with 15mM 1-NA-PP1 for 15 minutes on *KPNB1*. n=3 biological replicates, mean ± SEM, p-value: * $p < 0.05$, ** $p < 0.01$, *** $p < 0.001$. Statistical test: two-tailed unpaired t test.

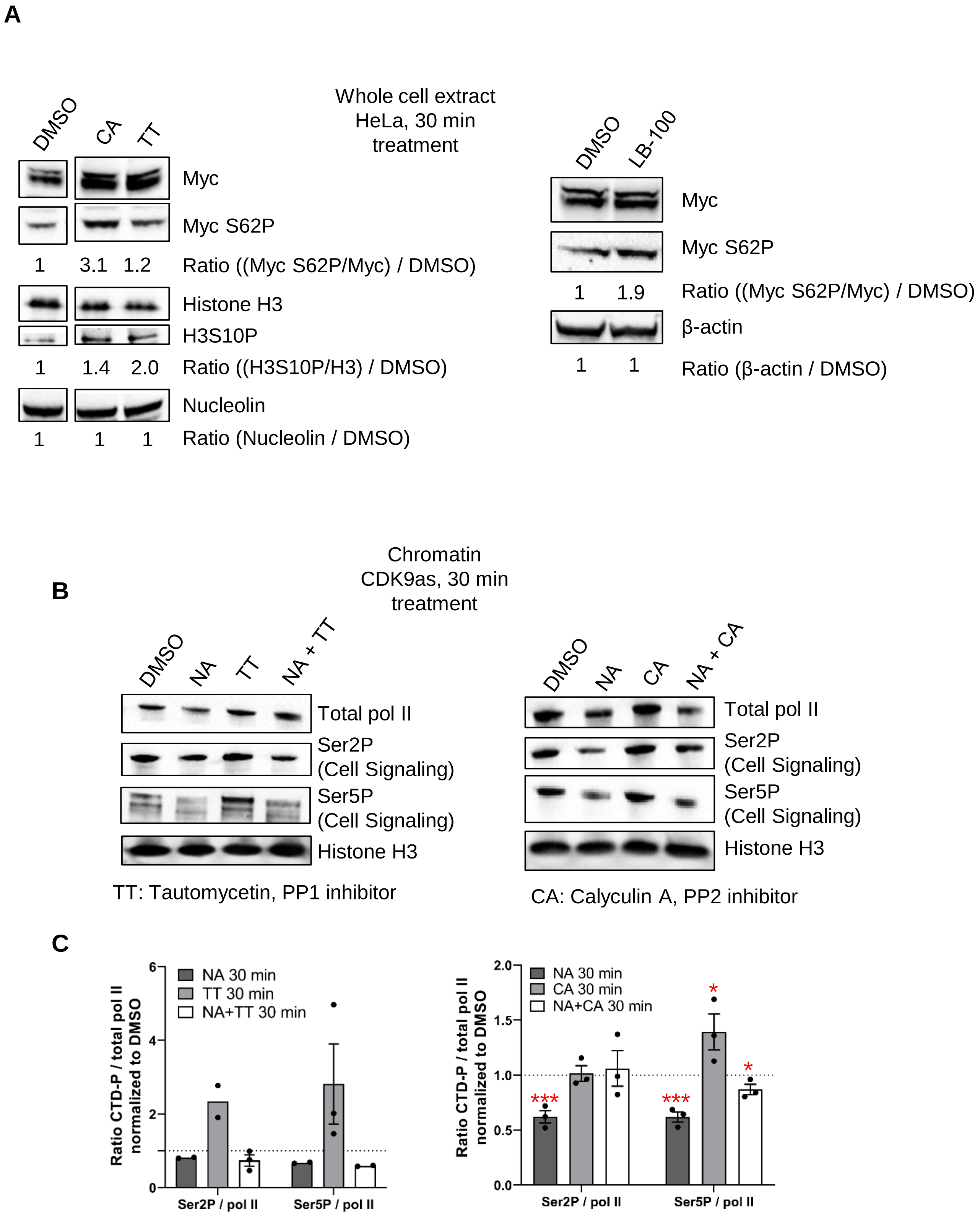

Appendix Figure S3

Appendix Figure S3. CDK9 phosphorylates several transcription and splicing factors *in vivo*.

A. Western blot of Myc, Myc S62P, Histone H3, Histone H3 S10P, and Nucleolin as a loading control, on whole cell extract of HeLa cells treated for 30 minutes with DMSO, DRB, CA, LB-100, or TT. **B.** Western blot of total pol II, Ser2P, Ser5P, and histone H3 as a loading control, on the chromatin fraction of CDK9as cells treated for 30 minutes with DMSO, NA, CA, TT, NA+CA, or NA+TT. The histone H3 loading control (CA samples) is the same as the loading control shown in Figure 4B as the same experiment is shown in two different figures. **C.** Quantification of the western blots shown in B. n=3 biological replicates except for Ser2P / pol II NA and TT and for Ser5P/ pol II NA and NA+TT where n=2 biological replicates, mean ± SEM, p-value: * $p < 0.05$, *** $p < 0.001$. Statistical test: two-tailed unpaired t test.

A

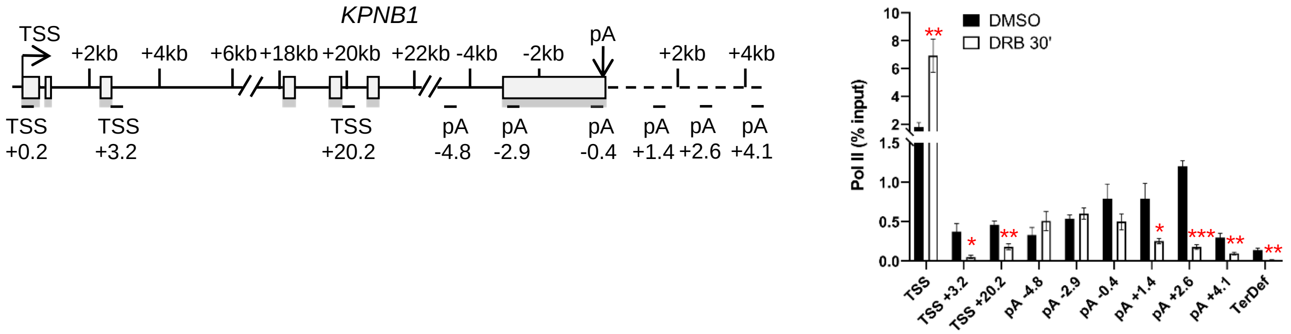

B

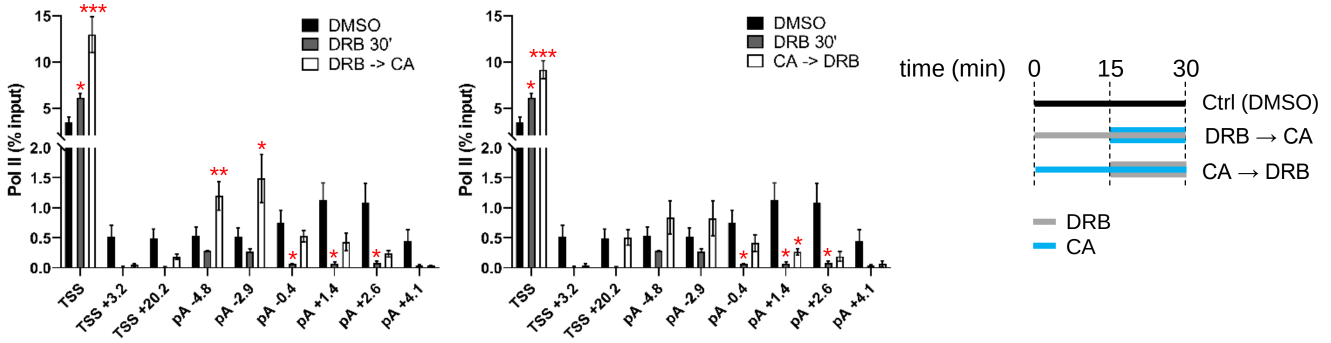

C

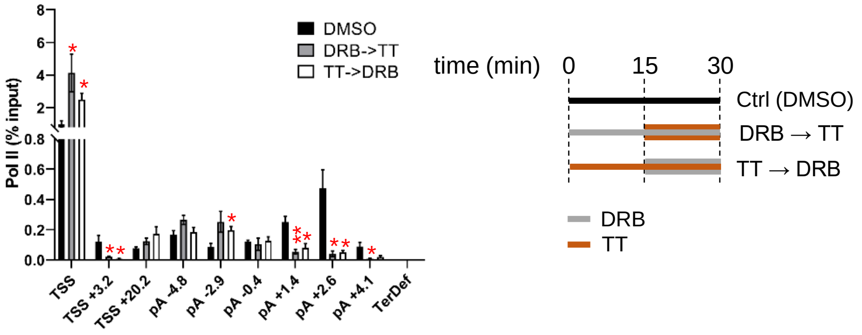

Appendix Figure S4

Appendix Figure S4. PP1 and PP2A regulate transcription of protein-coding genes.

A. ChIP-qPCR of total pol II after 30 minutes treatment with DMSO or DRB on *KPNB1*. n=3 biological replicates, mean ± SEM, p-value: * $p < 0.05$, ** $p < 0.01$, *** $p < 0.001$. Statistical test: two-tailed unpaired t test. **B.** ChIP-qPCR of total pol II after 30 minutes treatment with DMSO or DRB, or 15 minutes treatment with DRB or CA followed by another 15 minutes with CA (DRB->CA) or DRB (CA->DRB), respectively, on *KPNB1*. n=3 biological replicates, mean ± SEM, p-value: * $p < 0.05$, ** $p < 0.01$, *** $p < 0.001$. Statistical test: two-tailed unpaired t test. **C.** ChIP-qPCR of total pol II after 30 minutes treatment with DMSO or DRB, or 15 minutes treatment with DRB or TT followed by another 15 minutes with TT (DRB->TT) or DRB (TT->DRB), respectively, on *KPNB1*. n=3 biological replicates, mean ± SEM, p-value: * $p < 0.05$, ** $p < 0.01$, *** $p < 0.001$. Statistical test: two-tailed unpaired t test.

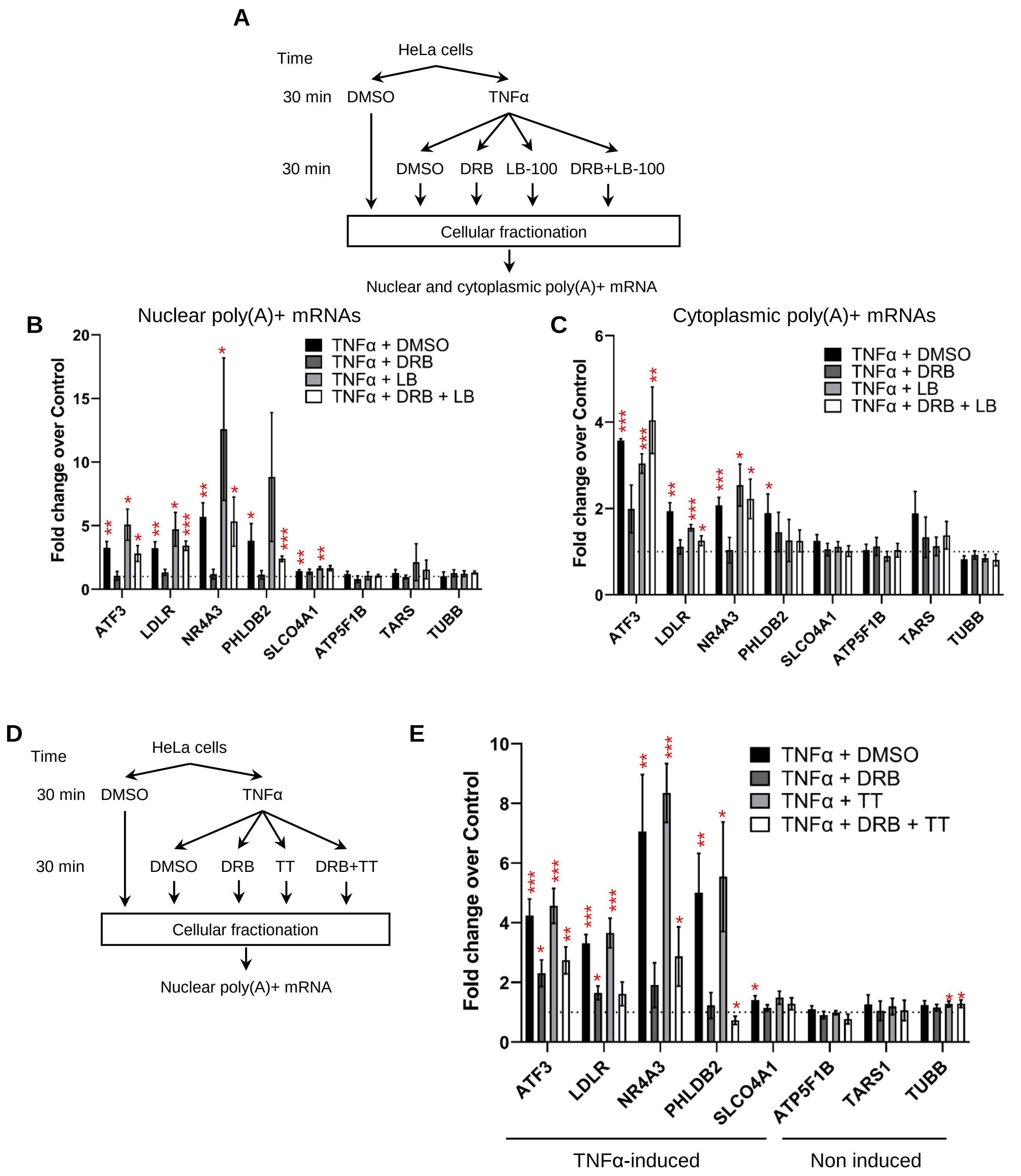

Appendix Figure S5

Appendix Figure S5. CDK9 and PP2A regulate mRNA cleavage and polyadenylation.

A. Schematic of the nuclear and cytoplasmic qRT-PCR experiments. **B.** qRT-PCR of nuclear polyadenylated mRNAs of several TNFα induced or non-induced genes with a 30 minutes DMSO, DRB, LB, or DRB+LB-100 treatment. n=3 biological replicates, mean ± SEM, p-value: * $p < 0.05$, ** $p < 0.01$, *** $p < 0.001$. Statistical test: two-tailed unpaired t test. **C.** qRT-PCR of cytoplasmic polyadenylated mRNAs of several TNFα induced or non-induced genes with a 30 minutes DMSO, DRB, LB-100, or DRB+LB-100 treatment. n=3 biological replicates, mean ± SEM, p-value: * $p < 0.05$, ** $p < 0.01$, *** $p < 0.001$. Statistical test: two-tailed unpaired t test. **D.** Schematic of the nuclear qRT-PCR experiments. **E.** qRT-PCR of nuclear polyadenylated mRNAs of several TNFα induced or non-induced genes with a 30 minutes DMSO, DRB, TT, or DRB+TT treatment. n=4 biological replicates, mean ± SEM, p-value: * $p < 0.05$, ** $p < 0.01$, *** $p < 0.001$. Statistical test: two-tailed unpaired t test.

Appendix Table S1: List of antibodies used in mNET-seq, ChIP, co-immunoprecipitation, and western blots.

Antibody	Supplier	Catalogue number	Applications
Mouse monoclonal anti-Pol II CTD, Total	MBL international	MABI0601	mNET-seq
Rpb1 NTD (D8L4Y) Rabbit mAb	Cell Signaling Technology	14958S	ChIP, ChIP-seq, Western blot
Phospho-Rpb1 CTD (Ser2) (E1Z3G) Rabbit mAb	Cell Signaling Technology	13499S	ChIP, Western blot
Phospho-Rpb1 CTD (Ser5) (D9N5I) Rabbit mAb	Cell Signaling Technology	13523S	ChIP, Western blot
RNA pol II antibody (mAb)	Active Motif	39097	Co-immunoprecipitation
Anti-RNA polymerase II CTD repeat YSPTSPS (phospho S2) antibody	Abcam	ab5095	Western blot
Anti-RNA polymerase II CTD repeat YSPTSPS (phospho S5) antibody	Abcam	ab5131	Western blot
Rabbit anti-SF3b155/SAP155 Antibody, Affinity Purified	Bethyl Laboratories	A300-996A	Western blot
SF3b1 T142P	This study	/	Western blot
Anti-SAP155 Monoclonal Antibody	MBL international	D221-3	Co-immunoprecipitation
Rabbit anti-SF3B3 Antibody, Affinity Purified	Bethyl Laboratories	A302-508A	Western blot
Myc	Santa Cruz Biotechnology	sc-764X	Western blot
Myc S62P	Abcam	ab51156	Western blot
Anti-Cdk9 antibody [EPR3119Y]	Abcam	ab76320	Western blot
Cyclin T1 (H-245)	Santa Cruz Biotechnology	sc-10750	Western blot
Rabbit anti-CPSF30 Antibody, Affinity Purified	Bethyl Laboratories	A301-584A	ChIP, Western blot
Rabbit anti-CPSF73 Antibody, Affinity Purified	Bethyl Laboratories	A301-091A	ChIP, Western blot
Rabbit anti-CPSF100 Antibody, Affinity Purified	Bethyl Laboratories	A301-581A	ChIP, Co-immunoprecipitation, Western blot
Rabbit anti-PAPOLA Antibody, Affinity Purified	Bethyl Laboratories	A301-008A	ChIP, Western blot
Rabbit anti-XRN2 Antibody, Affinity Purified	Bethyl Laboratories	A301-103A	ChIP, Western blot
Histone H3	Abcam	ab1791	Western blot
Histone H3 S10P	Life Technologies	PA517869	Western blot
Nucleolin	Abcam	ab22758	Western blot
Beta-actin	Cell Signaling Technology	4967S	Western blot
Anti-beta Tubulin antibody - Loading Control	Abcam	ab6046	Western blot
Normal Rabbit IgG	Cell Signaling Technology	2729S	ChIP, Co-immunoprecipitation

Appendix Table S2: List of primers used for ChIP-qPCR and qRT-PCR

Name	Forward primer	Reverse primer
**ChIP primers**		
KPNB1 TSS	TTACTTCCTCCCTCCAAATGGG	ACAGCCTCCCTTCCTTCTTTC
KPNB1 TSS+3.2	GCCCAGAGAACAAGAAATCG	GGAATGGACAAGCTGTGTTG
KPNB1 TSS+20.2	TGCAAGAGCCAGTGGGAACACTT	CCTCTACTCAGCAATGATACTTC
KPNB1 pA-4.8	CTGAGGAAACTGAAGAACCAAG	GAAGGCAGTGCTTGCCAGAAT
KPNB1 pA-2.9	GAGGAGTGTGCACGGATGCTGAA	CCAAGATGGCCGATGTTATGG
KPNB1 pA-0.4	TAGTTACCGTCTGCTTGGGAAGATG	CCTCTGACAGCAAGTCCAACATT
KPNB1 pA+1.4	GACTCATCACACCAAGGTCAC	GATAGTGCTGGGAAGGAAATGG
KPNB1 pA+2.6	GTACATCTCAGCTTTGGCATATG	GCCCAGAACATAGCAGGCATTGC
KPNB1 pA+4.1	GTTTCACCGTGTTAGCCAGGATGG	CCACAGCCATGTTCATTTCTGC
KPNB1 TD	AGGAGCATGGCTTTTCTCTG	TCATGCTGGAACTGGTTGAG
Neg	TGGTACAACCACAGCTCAGTG	AAGCTGGACATGGTTGTGTG
LDLR TSS	AATCACCCCACTGCAAACTC	TAGCTGGAAACCCTGGCTTC
LDLR TSS +1.9	TGGGATTGCCTGATGAACAC	AAGGCAGTTCAAAGCTCTCC
LDLR TSS +25	TTCAGTGTGGTGCTGACAAC	TTCTCTGCTGGAAACCCAAC
LDLR pA -1.8	CAGAGAAGACCAAAGCATTGCC	AATCCCAACCCAAGCCATTG
LDLR pA	AATCGCCGTGTTACTGTTGC	TGCCAATCCCTTGTGACATC
LDLR pA +1.6	GTGATTGTGTTCTCTGCTGTCG	TCGCACTTAGCACTCAACAG
**qRT-PCR primers**		
ATF3	AACCACAGTCAGTGGAGAGATG	TTCTCACAGCTGCAAACACC
LDLR	TTTGACGGGACTTCAGGTTC	TCCCTTGTGACATCTTCACG
NR4A3	AAGCCACCAGCTGTTAATGG	GCAATGCTGTTAGAGGAGCAG
PHLDB2	GAAACGACTTCAGGCAAGTCTC	GCCATGTTTTAGGAAAGAGCAC
SLCO4A1	TCCTCTTCTTTGCCATAGCC	ACAAGTTTCCAGGCCATCTG
ATP5F1B	ACCCATTGAAGAAGCTGTGG	CAATCAAGGCTCTTGTGCAG
TARS1	CATGGAAAAGGAGGAACAGC	CTTTGCCAAACTCCTCCAAG
TUBB	ATATGTTCCTCGTGCCATCC	TTTGGCCCAGTTGTTACCTG
GAPDH	CAACGACCACTTTGTCAAGC	TTCCTCTTGTGCTCTTGCTG
EIF1 distal pA	GCCTGAAACCAAGCAATACC	GTTCCGGCCATAGTTGTTTG
HCCS distal pA	CTCGAAAAGCCTGAACAACC	TCTTGAGCATCACCATGGAC
PCF11 distal pA	TGGAATTGAACAGCAACCTC	GAACCCCCTTTTGAAGGATG
EIF1 total	AGGGATCGCTGATGATTACG	TCTCCATATTCCGGATGCTC
HCCS total	TGGTGGTGAAGTCAACAAGG	CCACCAAGCGACTTTCATTC
PCF11 total	TCCACTCCTCCAATTGTTCC	AGCTCCAGCTTTTTCTGCTG
