# Supplementary material for: CDK9 and PP2A regulate RNA polymerase II transcription termination and coupled RNA maturation
Source: EMBO Rep. 2022 Aug 18;23(10):e54520. doi: 10.15252/embr.202154520 (PMC9535751; doi:10.15252/embr.202154520)
Supplement: Supplementary file 2 — Expanded View Figures PDF [file EMBR-23-e54520-s006.pdf]

Expanded View Figures

Figure EV1. CDK9 inhibition causes an elongation defect starting at the last exon of protein-coding genes.

A Metagene profile of total pol II after treatment with DMSO (black) or 5 (red), 10 (orange), or 15 (purple) minutes with DRB (green) around the TSS of expressed protein-coding genes (n = 6,965).

B Metagene profile of total pol II after treatment with DMSO (black) or 5 (red), 10 (orange), or 15 (purple) minutes with DRB (green) around the TSS of expressed protein-coding genes longer than 40 kb (n = 2,816).

C ChIP-qPCR of total pol II with different treatment times or different concentrations of DRB on *KPNB1*. n = 3 biological replicates, mean ± SEM, P-value: *$P < 0.05$, **$P < 0.01$, ***$P < 0.001$. Statistical test: two-tailed unpaired t-test.

D Metagene profile of total pol II after 30-min treatment with DMSO (black) or DRB (green) around internal exons, not including first, penultimate or last exons, of expressed protein-coding genes (n = 26,094).

© 2022 The Authors

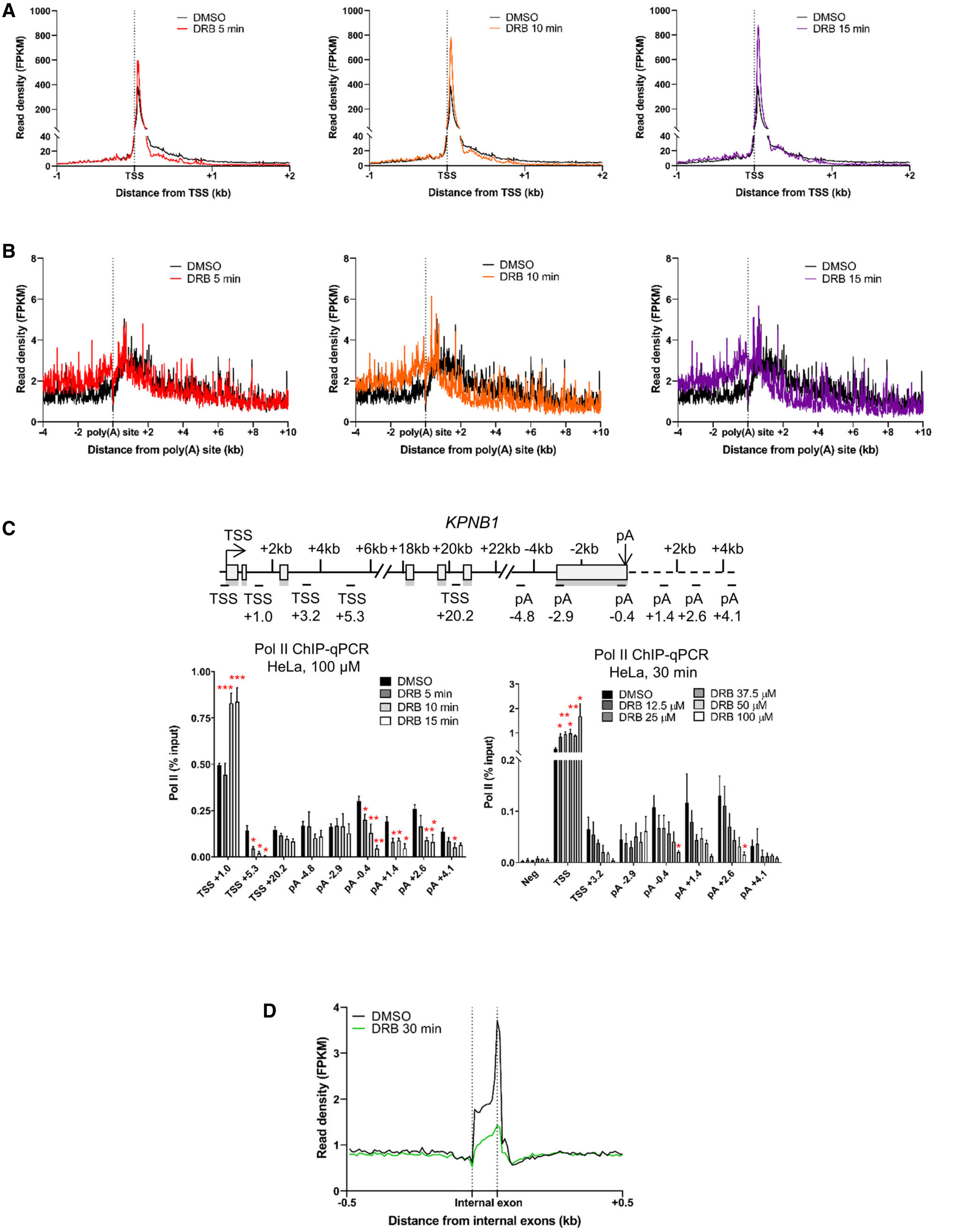

Figure EV1.

 © 2022 The Authors

Figure EV2. Inhibition of analog-sensitive (as) CDK9 produces similar results to small molecule CDK9 inhibitors.

A Schematic of the genome editing of the CDK9as cell line.
B alamarBlue cell viability of wild-type HEK293 and CDK9as cells. Each line represents a biological replicate.
C alamarBlueHS cell viability assay of wild-type HEK293 and CDK9as cells with different concentrations of 1-NA-PP1 added at the 36-h time point. $n = 3$ biological replicates, P-value: $^{**}P < 0.01$, $^{***}P < 0.001$. Statistical test: paired t-test with FDR multiple testing correction.
D ChIP-qPCR of total pol II with different concentrations of 1-NA-PP1 in CDK9as cells on *KPNB1*. $n = 3$ biological replicates, mean ± SEM, P-value: $^{*}P < 0.05$, $^{***}P < 0.001$. Statistical test: two-tailed unpaired t-test.
E ChIP-qPCR of total pol II treated with 1-NA-PP1 in wild-type HEK293 cells on *KPNB1*. $n = 3$ biological replicates, mean ± SEM, P-value: n.s. not significant. Statistical test: two-tailed unpaired t-test.
F Western blot of CDK9, Cyclin T1, and β-tubulin as a loading control, on whole-cell extracts of wild-type HEK293 and the CDK9as cell line treated with DMSO or 1-NA-PP1 for 15 or 30 min.
G Quantification of the Western blots shown in F. $n = 3$ biological replicates, mean ± SEM, P-value: not significant. Statistical test: two-tailed unpaired t-test.
H qRT–PCR of nuclear polyadenylated mRNAs of several TNFα-induced or noninduced genes with a 30-min DMSO or a NA treatment. $n = 3$ biological replicates, mean ± SEM, P-value: not significant. Statistical test: two-tailed unpaired t-test.

Source data are available online for this figure.

© 2022 The Authors

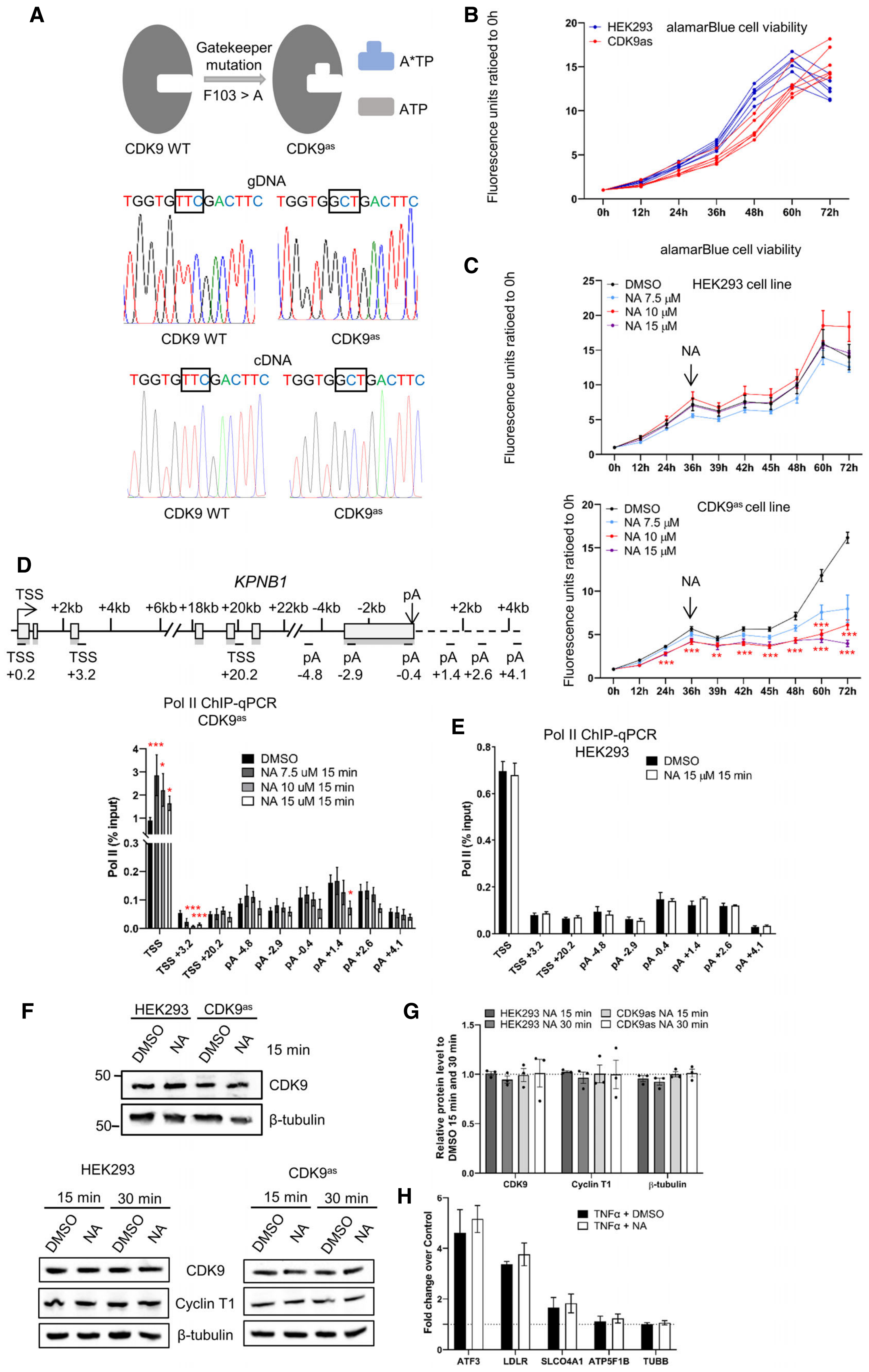

Figure EV2.

 © 2022 The Authors

Figure EV3. CDK9 phosphorylates several transcription and splicing factors *in vivo*.

A Volcano plot of SILAC phosphoproteomics in HeLa cells treated or not with 100 μM DRB for 30 min (in red: fold change > 1.5 in both biological duplicates, *P*-value < 0.1).

B Motif found around all the phosphorylation sites decreased following CDK9 inhibition of only the phosphorylation sites containing a ST or TP sites.

C Overlap between the proteins found to have at least one phosphopeptides decreased in our study versus an alternative experimental strategy used to identify CDK9 targets in cell extracts (Sanso *et al*, 2016).

D Quantification of the Western blots shown in Fig 4B. $n = 2$ biological replicates for the TT set, $n = 3$ biological replicates for the CA set, mean ± SEM, *P*-value: $^{**}P < 0.01$, $^{***}P < 0.001$. Statistical test: two-tailed unpaired *t*-test.

E Western blot of SF3B1 and β-actin, as a loading control, on the whole-cell extract of CDK9as cells after 30-min DMSO, NA, CA, or NA + CA treatment, or DMSO, NA, TT, NA + TT treatment.

Source data are available online for this figure.

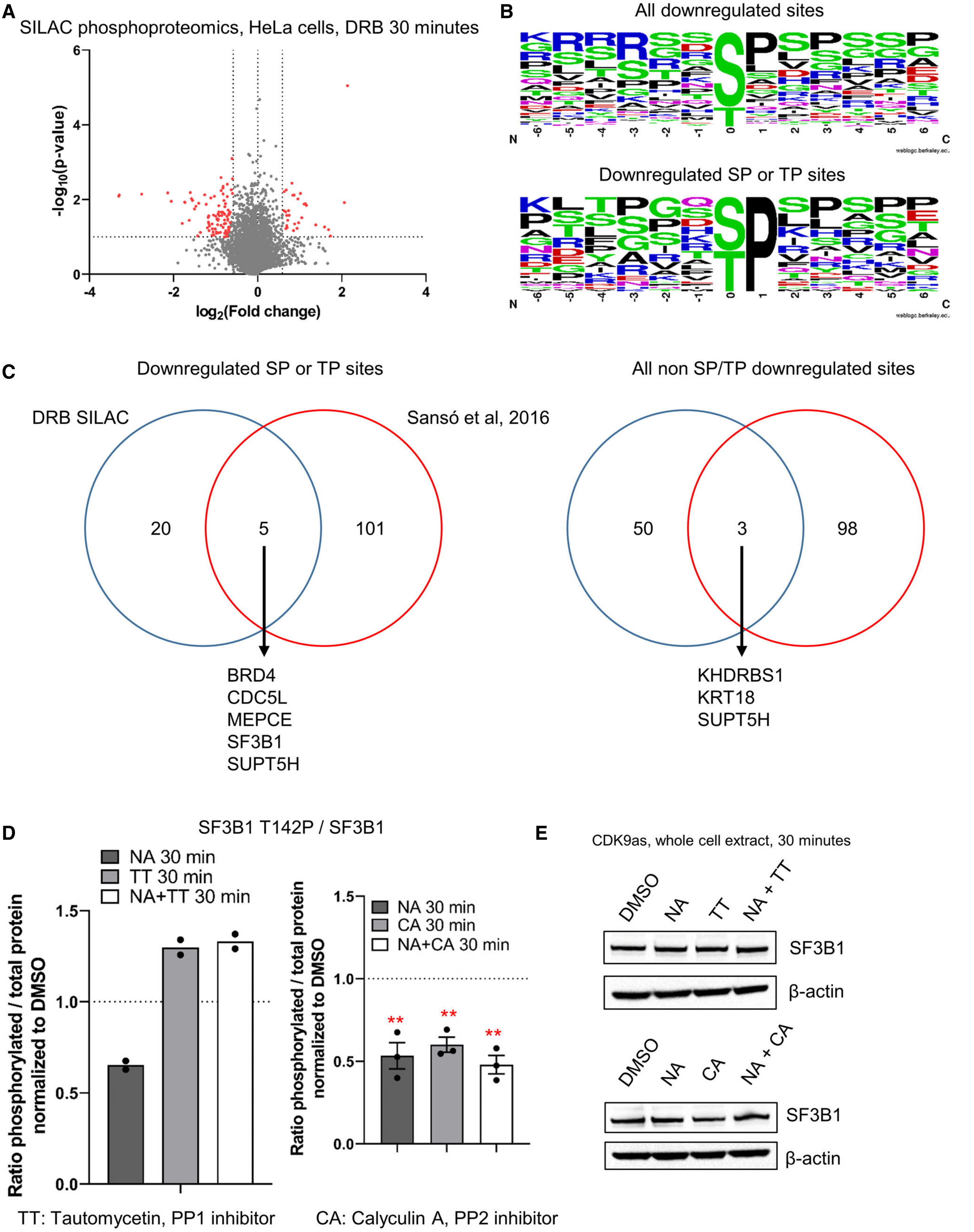

Figure EV3.

 © 2022 The Authors

Figure EV4. CDK9 and PP2A regulate mRNA cleavage and polyadenylation.

A Western blot of Xrn2, CPSF2, CPSF73, and histone H3 as a loading control, on the chromatin fraction of HeLa cells after 30-min DMSO, DRB, CA, or DRB + CA treatment.

B Quantification of the Western blots shown in (C). n = 4 biological replicates for DRB 30 min, n = 3 biological replicates for Xrn2 CA 30 min and DRB + CA 30 min, CPSF2 CA 30 min, and CPSF73 DRB + CA 30 min, n = 2 biological replicates for CPSF73 CA 30 min and CPSF2 DRB + CA 30 min, mean.

C Western blot of Xrn2, CPSF2, and Nucleolin as a loading control, on the nucleoplasm fraction of HeLa cells after a 30 min DMSO, DRB, CA, or DRB + CA treatment. The CPSF73 antibody does not provide reliable results on the nucleoplasm fraction.

D Western blot of total pol II, Ser5P, Xrn2, CPSF2, CPSF73, and β-tubulin as a loading control, on whole-cell extract of HeLa cells treated for 30 min with DMSO, DRB, CA, or DRB + CA.

E ChIP-qPCR of pol II, Ser2P, CPSF73, or CPSF2 after 30-min treatment with DMSO, DRB, CA, or DRB + CA on *KPNB1*. n = 3 biological replicates, mean ± SEM, P-value: *$P < 0.05$, **$P < 0.01$, ***$P < 0.001$. Statistical test: two-tailed unpaired t-test.

F qRT–PCR of nuclear polyadenylated mRNAs of the KPNB1 gene with a 30-min DMSO, DRB, TT, CA, DRB + TT, or DRB + CA treatment. n = 4 biological replicates, mean ± SEM, P-value: *$P < 0.05$, **$P < 0.01$, ***$P < 0.001$. Statistical test: two-tailed unpaired t-test.

Source data are available online for this figure.

© 2022 The Authors

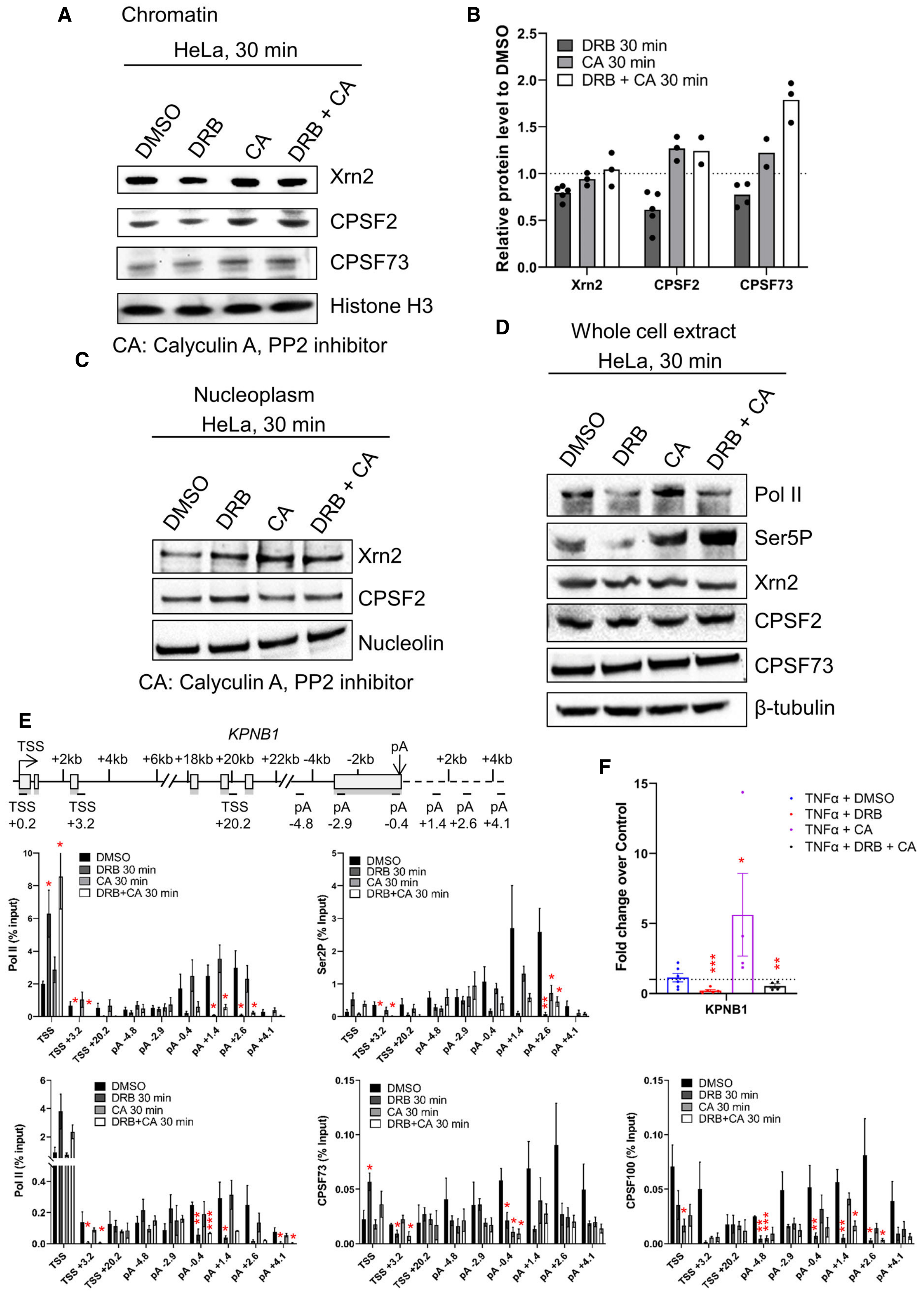

Figure EV4.

 © 2022 The Authors

Figure EV5. CDK9 and PP2A are involved in mRNA alternative poly(A) site usage.

A Number of genes undergoing significant increased or decreased intronic poly(A) site usage in both biological replicates of the 3′READS experiments.

B Number of genes undergoing significant increased or decreased proximal poly(A) site usage in both biological replicates of the 3′READS experiments.

C Screenshots of the genome browser 3′READS tracks at the 3′end of protein-coding genes EIF1, HCCS, and PCF11, which are undergoing decreased poly(A) site usage following CDK9 inhibition with DRB (indicated by the arrow).

D qRT–PCR of nuclear polyadenylated mRNAs of the EIF1, HCCS, and PCF11 genes with a 30-min DMSO, DRB, CA, or DRB + CA treatment. Two pairs of primers were used for each gene, one pair for the total transcripts level and one pair specific for the transcript using the distal poly(A) site usage. The data are shown as distal poly (A) site / total and normalized to the DMSO control. A value below 1 corresponds to a shift to proximal poly(A) site usage while a value superior to 1 corresponds to an increased distal poly(A) site usage. $n = 3$ biological replicates for the TNFα + DRB EIF1 and HCCS genes, $n = 4$ biological replicates for the TNFα + DRB EIF1 and HCCS genes and for all TNFα + DRB + CA genes, $n = 5$ biological replicates for the TNFα + DRB PCF11 gene, $n = 6$ biological replicate for the TNFα + CA PCF11 gene, mean ± SEM, *P*-value: $^{*}P < 0.05$, $^{***}P < 0.001$. Statistical test: two-tailed unpaired *t*-test.

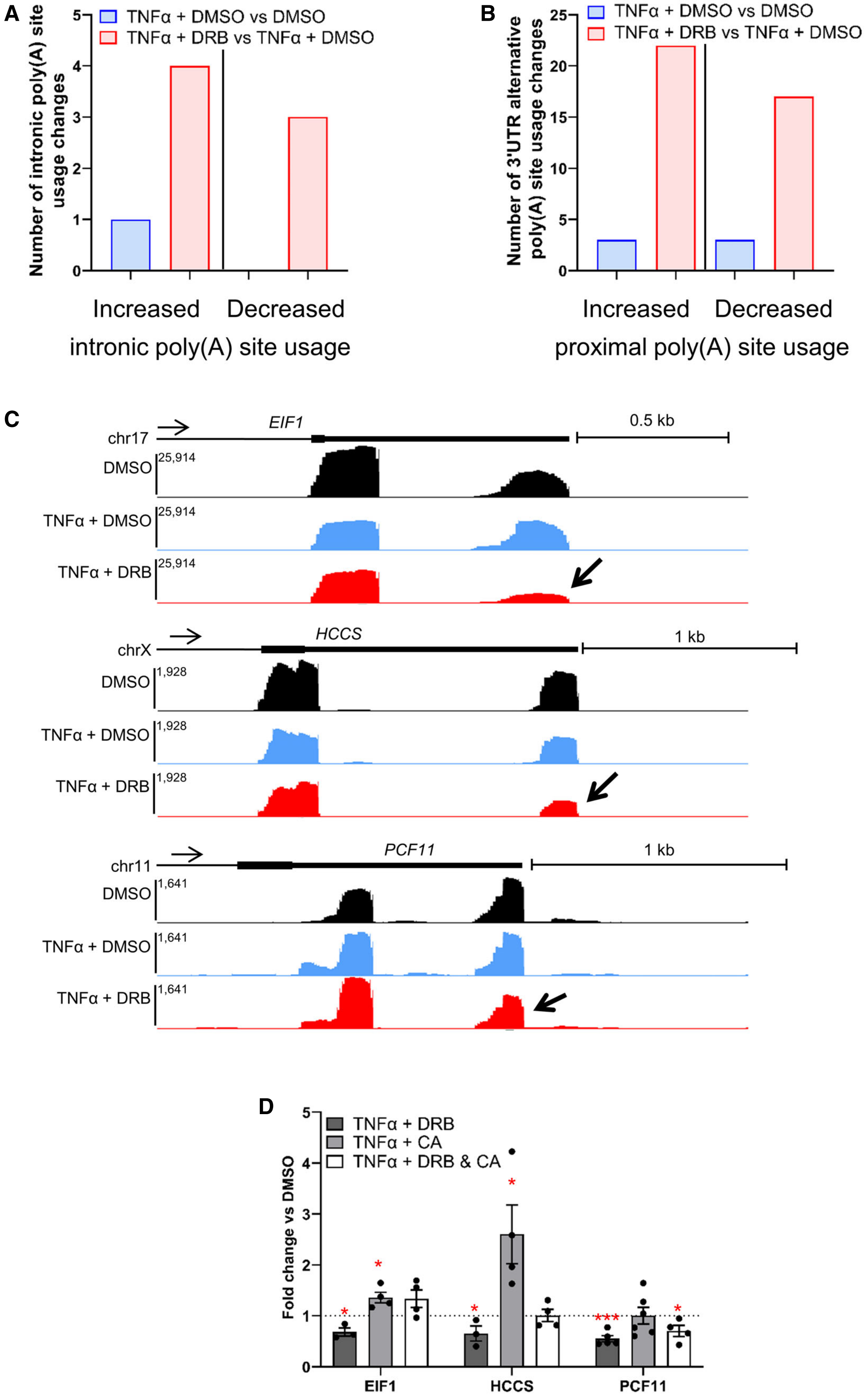

Figure EV5.

 © 2022 The Authors
