# Supplementary figures and images for: CDK9 and PP2A regulate RNA polymerase II transcription termination and coupled RNA maturation
Source: EMBO Rep. 2022 Aug 18;23(10):e54520. doi: 10.15252/embr.202154520 (PMC9535751; doi:10.15252/embr.202154520)

Appendix Figure S3A

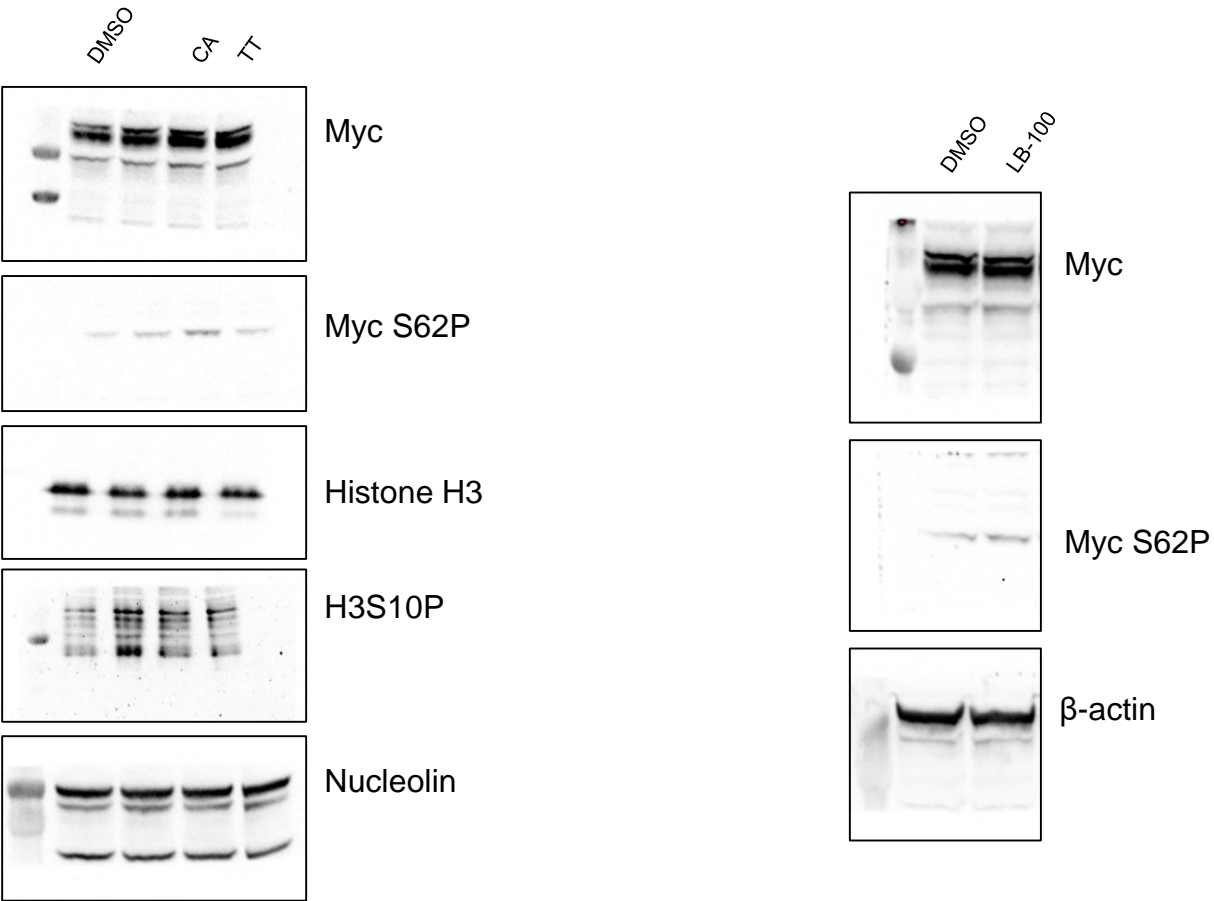

Appendix Figure S3B

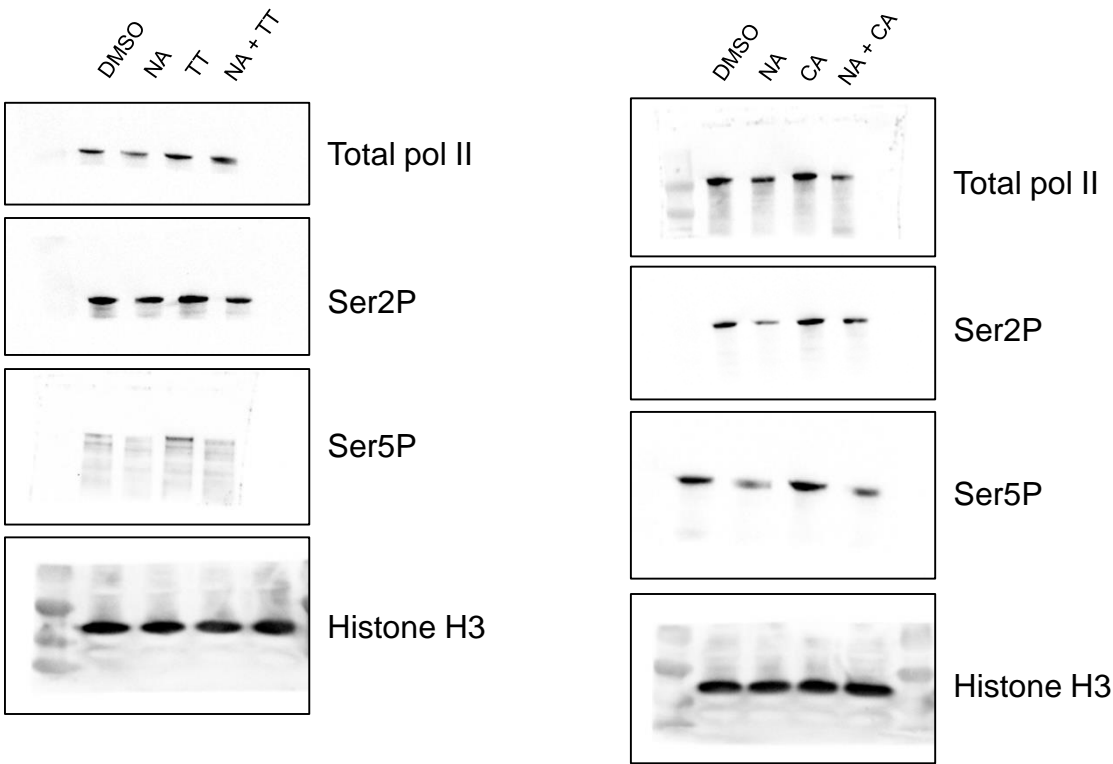

Appendix Figure S3

Supplement: Supplementary file 5 — Source Data for Expanded View and Appendix [file EMBR-23-e54520-s001.zip › EV_and_Appendix_Figure_Source_Data/Appendix_Figure_S3.pdf]

Appendix Figure S1C

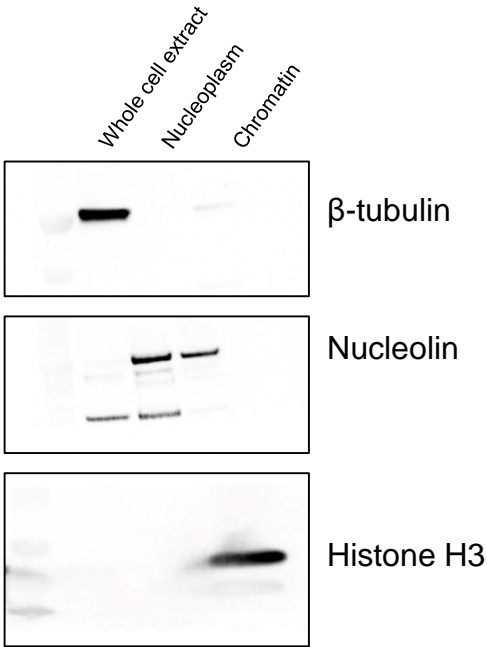

Appendix Figure S1D

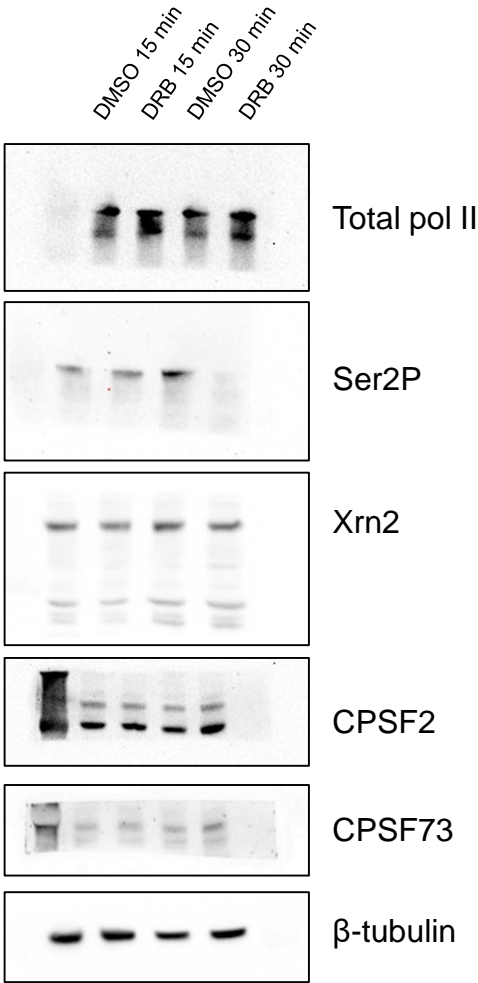

Appendix Figure S1

Supplement: Supplementary file 5 — Source Data for Expanded View and Appendix [file EMBR-23-e54520-s001.zip › EV_and_Appendix_Figure_Source_Data/Appendix_Figure_S1.pdf]

Figure EV4A

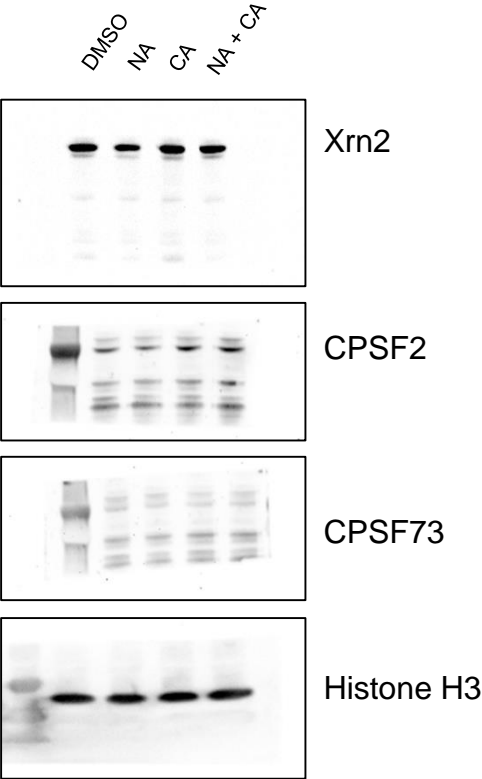

Figure EV4C

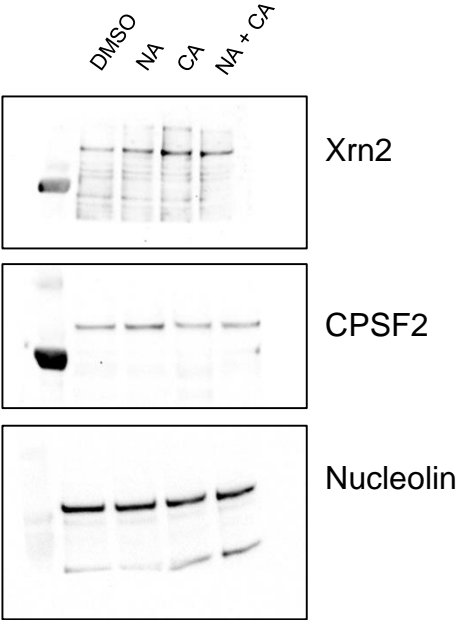

Figure EV4D

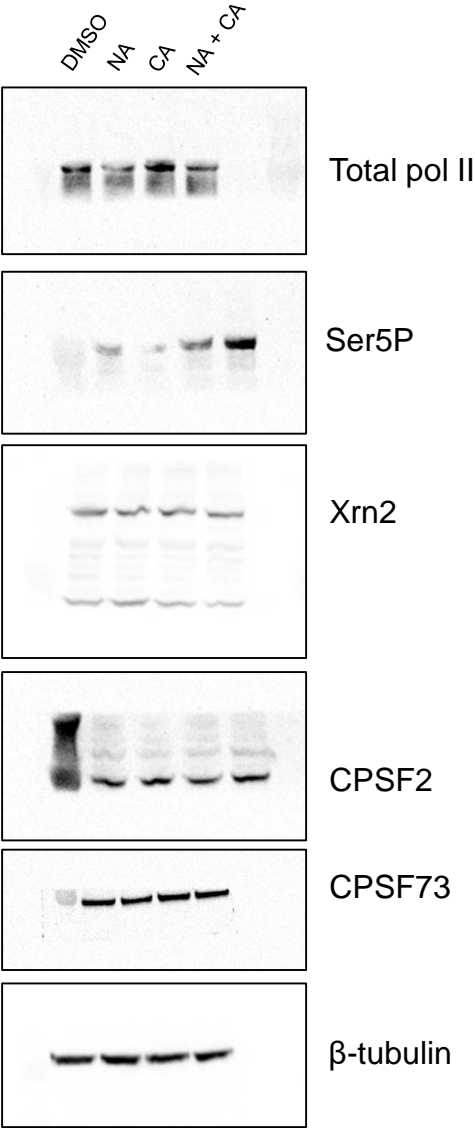

Figure EV4

Supplement: Supplementary file 5 — Source Data for Expanded View and Appendix [file EMBR-23-e54520-s001.zip › EV_and_Appendix_Figure_Source_Data/Figure EV4.pdf]

Figure EV2F

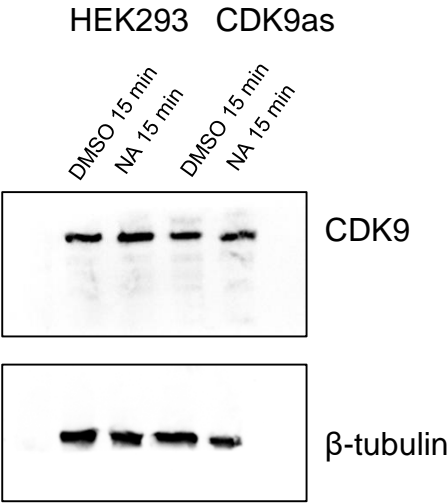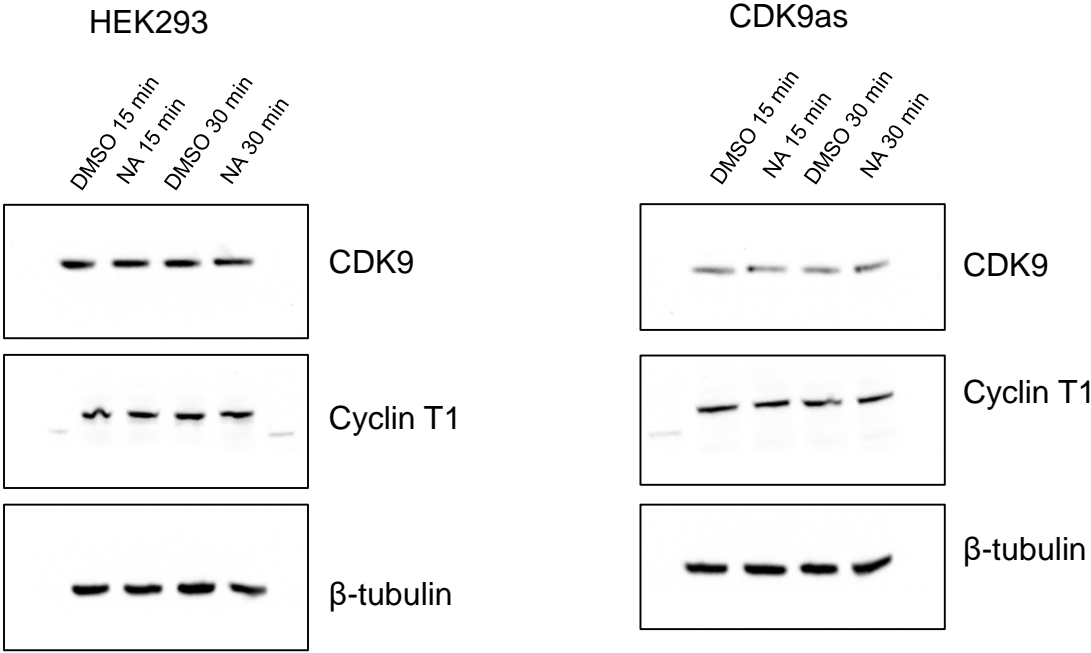

Figure EV2

Supplement: Supplementary file 5 — Source Data for Expanded View and Appendix [file EMBR-23-e54520-s001.zip › EV_and_Appendix_Figure_Source_Data/Figure EV2.pdf]

Figure EV3E

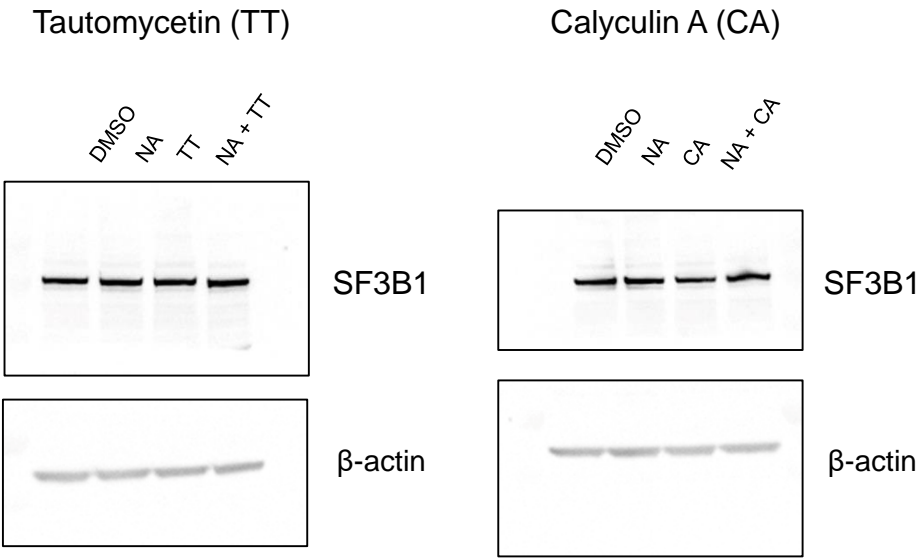

Figure EV3

Supplement: Supplementary file 5 — Source Data for Expanded View and Appendix [file EMBR-23-e54520-s001.zip › EV_and_Appendix_Figure_Source_Data/Figure EV3.pdf]
