# Supplementary material for: CDK9 and PP2A regulate RNA polymerase II transcription termination and coupled RNA maturation
Source: EMBO Rep. 2022 Aug 18;23(10):e54520. doi: 10.15252/embr.202154520 (PMC9535751; doi:10.15252/embr.202154520)
Supplement: Supplementary file 7 [file EMBR-23-e54520-s007.pdf]

EMBO reports

CDK9 and PP2A regulate RNA polymerase II transcription termination and coupled RNA maturation.

Michael Tellier, Justyna Zaborowska, Jonathan Neve, Takayuki Nojima, Svenja Hester, Marjorie Fournier, Andre Furger, and Shona Murphy
DOI: 10.15252/embr.202154520

Corresponding author(s): Michael Tellier (michael.tellier@path.ox.ac.uk) , Shona Murphy (shona.murphy@path.ox.ac.uk)

Review Timeline:

Submission Date:	15th Dec 21
Editorial Decision:	6th Feb 22
Revision Received:	9th May 22
Editorial Decision:	8th Jul 22
Revision Received:	13th Jul 22
Accepted:	21st Jul 22

Editor: Bernd Pulverer

Transaction Report:

(Note: With the exception of the correction of typographical or spelling errors that could be a source of ambiguity, letters and reports are not edited. Depending on transfer agreements, referee reports obtained elsewhere may or may not be included in this compilation. Referee reports are anonymous unless the Referee chooses to sign their reports.)

Dear Dr. Tellier

Thank you for the submission of your manuscript to EMBO reports. We have now received the full set of referee reports as well as referee cross-comments which are all pasted below.

Premature termination of RNAPII is associated with loss of mRNA polyadenylation and reduced recruitment of CPA and termination factors. You now propose that:
- SFB1 phosphorylation by CDK9 blocs CPA factor recruitment to polA sites in the last exon
- PP2A inhibition rescues premature termination from CDK9i
- PP2A inhibition promotes more efficient cleavage and polyadenylation of transcripts in an undefined manner.

As you will see, all three referees acknowledge that the findings are a potentially interesting extension of your previous NSMB paper on the topic. However, some of the data is judged to be not compelling in its current form (e.g. fig 4B and SF3B1 phoshorylation; ref 1 &2). They request also concrete evidence for a direct role of SF3B1 phoshorylation by CDK9 in the process and of PP2A mediated de-phosphorylation, and they note these events have to be demonstrated to formally support the mechanism proposed (ref 1 & 2). Finally, ref 3 requests that the study should look at alternative and intronic polA. In addition, the referees suggest a number of important points that can be address by textural revision (including potentially conflicting data from the Kourzarides group (who were not involved as referees).
Note that while ref leaves it open if the SF3B1 phoshorylation data is to be addressed experimentally, in our view this point has to be developed experimentally, in particular in light of providing clear evidence for the model proposed and a sufficiently striking conceptual advance over your previous work.

I would thus like to invite you to revise your manuscript with the understanding that the referee concerns must be fully addressed either textually or experimentally as suggested above. Please respond to all referee concerns in a complete point-by-point response. Acceptance of the manuscript will depend on a positive outcome of a second round of review. It is EMBO reports policy to allow a single round of major revision only and acceptance or rejection of the manuscript will therefore depend on the completeness of your responses included in the next, final version of the manuscript.

We realize that it is difficult to revise to a specific deadline. In the interest of protecting the conceptual advance provided by the work, we recommend a revision within 3 months (9th May 2022). Please discuss the revision progress ahead of this time with the editor if you require more time to complete the revisions.

You can either publish the study as a short report or as a full article. For short reports, the revised manuscript should not exceed 27,000 characters (including spaces but excluding materials & methods and references) and 5 main plus 5 expanded view figures. The results and discussion sections must further be combined, which will help to shorten the manuscript text by eliminating some redundancy that is inevitable when discussing the same experiments twice. For a normal article there are no length limitations, but it should have more than 5 main figures and the results and discussion sections must be separate. In both cases, the entire materials and methods must be included in the main manuscript file.

IMPORTANT NOTE: we perform an initial quality control of all revised manuscripts before re-review. Your manuscript will fail this control and the handling will be DELAYED if the following APPLIES:
1) A data availability section providing access to data deposited in public databases is missing. If you have not deposited any data, please add a sentence to the data availability section that explains that.
2) Your manuscript contains statistics and error bars based on n=2. Please use scatter blots in these cases. Only present statistical evidence where appropriate and show the indivisual datapoints (see also ref reports).

When submitting your revised manuscript, please carefully review the instructions that follow below. Failure to include requested items will delay the evaluation of your revision.

1) a .docx formatted version of the manuscript text (including legends for main figures, EV figures and tables). Please make sure that the changes are highlighted to be clearly visible.

2) individual production quality figure files as .eps, .tif, .jpg (one file per figure). See https://wol-prod-cdn.literatumonline.com/pb-assets/embo-site/EMBOPress_Figure_Guidelines_061115-1561436025777.pdf for more info on how to prepare your figures.

3) We replaced Supplementary Information with Expanded View (EV) Figures and Tables that are collapsible/expandable online. A maximum of 5 EV Figures can be typeset. EV Figures should be cited as 'Figure EV1, Figure EV2" etc... in the text and their respective legends should be included in the main text after the legends of regular figures.

- For the figures that you do NOT wish to display as Expanded View figures, they should be bundled together with their legends in a single PDF file called *Appendix*, which should start with a short Table of Content. Appendix figures should be referred to in the main text as: "Appendix Figure S1, Appendix Figure S2" etc. See detailed instructions regarding expanded view here:

<https://www.embopress.org/page/journal/14693178/authorguide#expandedview>

- Additional Tables/Datasets should be labeled and referred to as Table EV1, Dataset EV1, etc. Legends have to be provided in a separate tab in case of .xls files. Alternatively, the legend can be supplied as a separate text file (README) and zipped together with the Table/Dataset file.

4) a .docx formatted letter INCLUDING the reviewers' reports and your detailed point-by-point responses to their comments. As part of the EMBO Press transparent editorial process, the point-by-point response is part of the Review Process File (RPF), which will be published alongside your paper.

5) a complete author checklist, which you can download from our author guidelines <https://www.embopress.org/page/journal/14693178/authorguide>. Please insert information in the checklist that is also reflected in the manuscript. The completed author checklist will also be part of the RPF.

6) Please note that all corresponding authors are required to supply an ORCID ID for their name upon submission of a revised manuscript (<https://orcid.org/>). Please find instructions on how to link your ORCID ID to your account in our manuscript tracking system in our Author guidelines <https://www.embopress.org/page/journal/14693178/authorguide#authorshipguidelines>

7) Before submitting your revision, primary datasets produced in this study need to be deposited in an appropriate public database (see https://www.embopress.org/page/journal/14693178/authorguide#datadeposition). Please remember to provide a reviewer password if the datasets are not yet public. The accession numbers and database should be listed in a formal "Data Availability" section placed after Materials & Method (see also https://www.embopress.org/page/journal/14693178/authorguide#datadeposition). Please note that the Data Availability Section is restricted to new primary data that are part of this study. * Note - All links should resolve to a page where the data can be accessed. *
If your study has not produced novel datasets, please mention this fact in the Data Availability Section.

8) We would also encourage you to include the source data for figure panels that show essential data. Numerical data should be provided as individual .xls or .csv files (including a tab describing the data). For blots or microscopy, uncropped images should be submitted (using a zip archive if multiple images need to be supplied for one panel). Additional information on source data and instruction on how to label the files are available at <https://www.embopress.org/page/journal/14693178/authorguide#sourcedata>.

9) Our journal also encourages inclusion of *data citations in the reference list* to directly cite datasets that were re-used and obtained from public databases. Data citations in the article text are distinct from normal bibliographical citations and should directly link to the database records from which the data can be accessed. In the main text, data citations are formatted as follows: "Data ref: Smith et al, 2001" or "Data ref: NCBI Sequence Read Archive PRJNA342805, 2017". In the Reference list, data citations must be labeled with "[DATASET]". A data reference must provide the database name, accession number/identifiers and a resolvable link to the landing page from which the data can be accessed at the end of the reference. Further instructions are available at https://www.embopress.org/page/journal/14693178/authorguide#referencesformat

10) Regarding data quantification (see Figure Legends: https://www.embopress.org/page/journal/14693178/authorguide#figureformat)

The following points must be specified in each figure legend:

- the name of the statistical test used to generate error bars and P values,

- the number (n) of independent experiments (please specify technical or biological replicates) underlying each data point,

- the nature of the bars and error bars (s.d., s.e.m.),

- If the data are obtained from n {less than or equal to} 2, use scatter blots showing the individual data points.

Discussion of statistical methodology can be reported in the materials and methods section, but figure legends should contain a basic description of n, P and the test applied.

- Please also include scale bars in all microscopy images.

11) The journal requires a statement specifying whether or not authors have competing interests (defined as all potential or actual interests that could be perceived to influence the presentation or interpretation of an article). In case of competing interests, this must be specified in your disclosure statement. Further information: https://www.embopress.org/competing-interests

We would also welcome the submission of cover suggestions, or motifs to be used by our Graphics Illustrator in designing a cover.

As part of the EMBO publication's Transparent Editorial Process, EMBO reports publishes online a Review Process File (RPF) to accompany accepted manuscripts. This File will be published in conjunction with your paper and will include the referee reports, your point-by-point response and all pertinent correspondence relating to the manuscript.

You are able to opt out of this by letting the editorial office know (emboreports@embo.org). If you do opt out, the Review Process File link will point to the following statement: "No Review Process File is available with this article, as the authors have chosen not to make the review process public in this case."

I look forward to seeing a revised form of your manuscript when it is ready. Please use this link to submit your revision:
https://embor.msubmit.net/cgi-bin/main.plex

Yours sincerely,

Bernd Pulverer

~~~~~~~~~~~~~~~~~~~~~~~~~~~~~~~~
Bernd Pulverer, Ph.D.
Chief Editor, EMBO Reports
EMBO
Meyerhofstrasse 1, D-69117 Heidelberg
Tel: +4962218891501
bernd.pulverer@embo.org
~~~~~~~~~~~~~~~~~~~~~~~~~~~~~~~~

Referee #1:

Authors' group has previously demonstrated that CDK9 inhibition leads to an elongation block at an early elongation checkpoint as well as premature termination of RNA polymerase II (RNAPII) (NSMB22 :396). In this manuscript, the authors further investigated the mechanism of premature termination by CDK9 inhibition. First, CDK9 inhibition causes premature termination at the last exon. The recruitment of cleavage/polyadenylation (CPA) factors to RNAPII was impaired after CDK9 inhibition. Inhibition of PP2A, but not PP1, rescued this phenotype. Phosphoproteomic analyses with specific CDK9 inhibition in cells expressing analogue-sensitive (as) CDK9 identified a splicing factor SF3B1 as a key substrate of CDK9. From these results, authors conclude CDK9 not only stimulates transcription elongation, but mediates transcription termination and RNA maturation. The presented results are intriguing, and the authors conclusions are generally well supported by the data. Authors' proposal of CDK9's "new" role on termination is very exciting. In particular, usage of asCDK9 provided a stricter specificity for CDK9 inhibition, significantly reassuring the data obtained with DRB, a rather promiscuous CDK inhibitor.

Specific Comments

1. Figs. 4B and 4C: The phosphorylation data with SF3b1 T142P are rather "subtle" to me, comparing the data with SPT5 T806P. In particular, in the Fig4B, left panel, NA did not seem to reduce the band for T142P as much as it did in the right panel, making it difficult to judge whether CA can reverse the phenotype.
2. This study newly identified SF3B1 as a substrate of CDK9. However, the role of SF3B1 phosphorylation in CDK9-dependent transcription was not explored further. Although this might be beyond the scope of the presented study, any implications about potential roles of SF3B1 phosphorylation (e.g. phospo-SF3b1 is more active) would be helpful particularly in terms of the involvement of PP1 in the CDK9-dependent transcription.
3. It is unclear, and particularly of interest, whether the PP2A inhibition directly or indirectly reverses the phenotype of CDK9 inhibition. In other words, is the balance between Ser2 phosphorylation by CDK9 and dephosphorylation by PP2A the mechanism by which more CPA factors are recruited to CTD after NA+CA treatment?
4. With the same reasoning, the model figure (Fig.8) is not very informative in my opinion.
5. While the fist Net-seq and 'bulk' gene analysis were done with TNF stimulation, other experiments (ChIP-RTqPCR, phosphoproteomics, WB etc) were conducted in steady-state conditions, which is bit confusing since TNF signalling does induce P-TEFb activity. Does TNF increase the phosphorylation of SPT5 and/or SF3B1 or transcription of KPNB1, for example?
6. Other CDKs/Cyclins, including CDK12/CycK (GD28:342, MCB35:468), and CDK11/CycL(CHM 18:560), were also reported to mediate CPA. It would be helpful to include involvement of these studies in the Discussion.
7. Also, Kouzarides and colleagues demonstrated that hyperactivation of P-TEFb (by eliminating 7SKsnRNA by antisense oligos) causes global readthrough of the CPA sites (Genome Biol.14:R98). It seems that both CDK9 activation and inhibition lead to a defect in transcriptional termination. Further discussion about this would be helpful to visualise "a big picture" of CDK9-

mediated transcription control.

Referee #2:

This study is on CDK9 functions in gene expression with a focus on RNA polyadenylation and termination. Murphy and colleagues previously suggested that CDK9 inhibition causes premature termination of pol II transcripts (Laitem eta l., 2015). This was generally confirmed and extended with respect to CDK9 functions in transcription/processing factor occupancy at 3' regions of genes. The perhaps most innovative aspect of the paper relates to possible roles of phosphatases and their interplay with CDK9. In brief, the authors propose a novel role of PP2A in 3' end formation. Along these studies also CDK9 kinase targets have been reinvestigated.

This investigation is of interest and technically state of the art. Data density makes it in some parts difficult to read though. The reader is often left alone in deciding about the relevance of data and significance of conclusions. There remain some questions about data and conclusions.

1) One question concerns the definition of "premature termination" and "decreased polyadenylation". It is my understanding that these terms are exchangeable and the latter does not refer to downregulation of ´the process of polyadenylation. 3'READS technology measures polyadenylated 3' termini but does it in the end allow to discriminate between reduced polyadenylation activity, mitigated RNA synthesis and premature termination (Figure 1A, suppl. Figure 1A)? Please discuss and state in the ms.
2) For the separation of chromatin and nucleoplasm it would be helpful if the exact protocol for the actually applied method for chromatin and nucleoplasm separations was added. Attempts to find the information led through a series of publications into the last Millenium w/o clear result. This applies to protein and RNA separations (Fig 1C/D). Relatedly, I was wondering where the ribosomes go in the separation protocol (and the RNA being translated in them).
3) Figure 1F/G: The reader is certainly somewhat lost in the complexity of data, especially the many data points in the model gene, out of which some are significant others apparently not. In my view, it would be generally important to highlight(state statistical significance for those values that are critical to the conclusions. Also, data bars are often too low to visually judge about them. It appears for example i) that the decline of pol II at +5.3 is not much different from the decline in downstream pA regions (except for pA -4.8) and ii) that CPFs drop similar to pol II at various positions in the gene. I assume that the intention is to exactly show that his is not the case by plotting ratios of CPFs to pol II. Also, the normalization procedure is unclear. How can the ratio of Xrn2/pol II be more sensitive to DRB, than Xrn2 alone at pA 4.1, if pol II shows similar sensitivity as Xrn2 at this position?
4) The impact of the bulky adenosine-derivative (NA) on Ser2P-Cell signaling (Fig 3A) in HEK293 appears - at least visually - as strong as in CDK9as, relative to total pol II. Also the Ser5P values seem quite comparable between the sensitive and the insensitive control lines. This is in apparent contrast to the quantitative evaluation (Fig 3B). This is important as much of the following data is based on the CDK9as cell line. It would be helpful to have blots that support the final conclusion. Please change and/or clarify.
5) Figure 4B: SFB3b1 data is convincing, but can't see the effect of CDK9 blockage on SPT5-P806T. In fact, relative to total SPT5, NA seems to cause less of a decline and NA+TT is of low quality (left panel). Please clarify/exchange data!
6) Figure 7B: the authors state PP2A inhibition "fully reverses the effect of CDK9 inhibition". It is indeed intriguing that the DRB+CA levels are quite similar to the respective control values. However, how can the authors distinguish reversal at 3' ends from additive effects over the entire gene? Please discuss.

Minor:

1) What are the authors referring to with "Alternatively,..." in the third sentence of the result section?
2) It wasn't clear to me why the authors use a rather complex KPNB1 gene that apparently harbors a collection of polyA sites, with the pA at -0.4 being the prominent one, if not entirely for historical reasons? Please explain.
3) I don't think SNS-032 is a specific for CDK9 inhibitor (p8 and supp. Fig 2c). I would thus leave the data out.
4) Much is the based on the specificity of bulky adenosine in combination with the mutated kinase. It would be good to know whether NA has been tested on a broad panel of kinases. If so please quote.
5) The need of the proteomics data for the subsequent PP story isn't obvious to me. In fact, the authors continue with a phospho-site in SPT5 (806) that apparently wasn't even detected in their proteomics analysis. Certainly, the reader expects an explanation for the very small overlap with previous studies.
6) Figure 5: Data load is difficult to digest for the reader. Perhaps one could make them supplemental and limit data in the text to those that make the point.

Referee #3:

This manuscript by Tellier et al. reports novel functions of CDK9 and PP2A in 3' end processing/transcriptional termination. The work was based largely on using a set of CDK9 and PP2A inhibitors coupled with genome-wide sequencing. The key finding is that CDK9 inhibition leads to suppression of Pol II signals after the last poly(A) site and loss of CPA factors from chromatin, and these can be restored by co-inhibition of PP2A. The authors additionally showed that CDK9 can phosphorylate a group of factors that were not previously known to be its substrates, including SF3B1. The conclusions of this work are largely supported by their data. However, there are a few issues the authors need to address before the work can be accepted for publication.

1. A large number of polyA sites are located in introns. The authors should use their 3'READS data to examine their regulation.
2. For polyA sites in the last exons, the authors should examine whether there is alternative polyadenylation after CDK9 and PP2A inhibition. Based on their model, the alternative polyadenylation changes should be substantial. If so, the authors should also check if the regulation is related to pol II signal changes after the last polyA site, which is indicative of CPA defect.

Please find our responses to your and the reviewers' comments below. The changes are marked in the text.

Your comments

Premature termination of RNAPII is associated with loss of mRNA polyadenylation and reduced recruitment of CPA and termination factors. You now propose that:
- SFB1 phosphorylation by CDK9 blocs CPA factor recruitment to polA sites in the last exon

We are not entirely sure what you mean here-we initially proposed that SF3B1 phosphorylation by CDK9 is helping CPA recruitment and have now shown that inhibition of CDK9 leads to loss of SF3B1 in complex with CPA factors from pol II.

- PP2A inhibition rescues premature termination from CDK9i

Yes
- PP2A inhibition promotes more efficient cleavage and polyadenylation of transcripts in an undefined manner.

Yes and we have now shown that PP2A inhibition increases SF3B1 association with pol II, which could therefore promote better recruitment/activity of the mRNA cleavage and polyadenylation complex.

As you will see, all three referees acknowledge that the findings are a potentially interesting extension of your previous NSMB paper on the topic. However, some of the data is judged to be not compelling in its current form (e.g. fig 4B and SF3B1 phoshorylation; ref 1 &2). They request also concrete evidence for a direct role of SF3B1 phoshorylation by CDK9 in the process and of PP2A mediate de-phosphorylation and note these have to be demonstrated to formally support the mechanism proposed (ref 1 & 2). Finally, ref 3 requests that the study should look at alternative and intronic polA. In addition, the referees suggest a number of important points that can be address by textural revision (including potentially conflicting data from the Kourzarides group (who were not involved s referees).
Note that while ref leaves it open if the SF3B1 phoshorylation data is to be addressed experimentally, in our view this point has to be developed experimentally, in particular in light of providing clear evidence for the model proposed and a sufficiently striking conceptual advance over your previous work.

Referee #1:

Authors' group has previously demonstrated that CDK9 inhibition leads to an elongation block at an early elongation checkpoint as well as premature termination of RNA polymerase II (RNAPII) (NSMB22 :396). In this manuscript, the authors further investigated the mechanism of premature termination by CDK9 inhibition. First, CDK9 inhibition causes premature termination at the last exon. The recruitment of cleavage/polyadenylation (CPA) factors to RNAPII was impaired after CDK9 inhibition. Inhibition of PP2A, but not PP1, rescued this phenotype. Phosphoproteomic analyses with specific CDK9 inhibition in cells expressing analogue-sensitive (as) CDK9 identified a splicing factor SF3B1 as a key substrate of CDK9. From these results, authors conclude CDK9 not only stimulates transcription elongation, but mediates transcription termination and RNA maturation. The presented results are intriguing, and the authors conclusions are generally well supported by the data. Authors' proposal of CDK9's "new" role on termination is very exciting. In particular, usage of asCDK9 provided a stricter specificity for CDK9 inhibition, significantly reassuring the data obtained with DRB, a rather promiscuous CDK inhibitor.

We thank the reviewer for the positive comments.

Specific Comments

1. Figs. 4B and 4C: The phosphorylation data with SF3b1 T142P are rather "subtle" to me, comparing the data with SPT5 T806P. In particular, in the Fig4B, left panel, NA did not seem to reduce the band for T142P as much as it did in the right panel, making it difficult to judge whether CA can reverse the phenotype.

We have performed new western blots for SF3B1 T142P. In addition, we have removed the SPT5 T806P data as this residue was not identified as a target in our phosphoproteomics analysis.

2. This study newly identified SF3B1 as a substrate of CDK9. However, the role of SF3B1 phosphorylation in CDK9-dependent transcription was not explored further. Although this might be beyond the scope of the presented study, any implications about potential roles of SF3B1 phosphorylation (e.g. phospo-SF3b1 is more active) would be helpful particularly in terms of the involvement of PP1 in the CDK9-dependent transcription.

It was previously shown that the U2 snRNP, including the SF3B proteins, could interact with the CPA complex to couple pre-mRNA splicing and 3’end processing (Kyburz, Friedlein et al., 2006). We have therefore tested whether the loss of SF3B1 phosphorylation and/or recruitment to the chromatin could be involved in the loss of the CPA complex from the chromatin we observed after CDK9 inhibition.

While we could not observe a strong interaction between SF3B1 and CPA factors when we pulled down SF3B1, we found that pull down of CPSF100 detects interaction with SF3B1 and SF3B3. Following CDK9 inhibition, we did not observe any change in the interaction between SF3B1 and the CPA factors but found that the interactions of both SF3B1 and CPA factors with total pol II were decreased, indicating that the whole SF3B-CPA complex is lost from the pol II complex following CDK9 inhibition, which could explain why CDK9 inhibition promotes a loss of mRNA cleavage and polyadenylation. Future work will be needed to determine whether phosphorylation of SF3B1 by CDK9 is directly needed for the interaction of the SF3B complex with pol II.

3. It is unclear, and particularly of interest, whether the PP2A inhibition directly or indirectly reverses the phenotype of CDK9 inhibition. In other words, is the balance between Ser2 phosphorylation by CDK9 and dephosphorylation by PP2A the mechanism by which more CPA factors are recruited to CTD after NA+CA treatment?

This is an excellent point. This reversal of the effect of CDK9 inhibition by CDK9 + PP2A inhibition can only be monitored for a short time as inhibition of both CDK9 and PP2A does not reverse the effect of CDK9 inhibition at the early elongation checkpoint, meaning that pol II will "run off" genes over time. Therefore, one of the main differences in this wave of transcription (starting before CDK9 inhibition and stopping with CDK9 inhibition) between CDK9 inhibition and CDK9+PP2A inhibition is the recruitment of the CPA complex to the pol II, allowing the production of poly(A)+ mRNAs. This suggests that at least one of the factors common to both CDK9 and PP2A needs to be phosphorylated and/or present for the CPA complex to be recruited to pol II.

We have now shown (Figure 7E) that in addition to CDK9 inhibition decreasing the interaction between SF3B1 and pol II, PP2A inhibition increases the interaction of SF3B1 with pol II. However, inhibition of PP2A and CDK9 together does not bring back SF3B1 interaction with pol II to the control level (DMSO), which is in contrast with CPA factors that are interacting with pol II at the control level or higher. This indicates that in the CDK9 + PP2A inhibition condition, SF3B1 is not likely to be the common factor. Phosphorylation of Ser2 of the pol II CTD is involved in transcription elongation and termination and in mRNA CPA (Davidson, Muniz et al., 2014, Eifler, Shao et al., 2015, Tellier, Zaborowska et al., 2020). In addition, it is one of the common targets of CDK9 and PP2A, which makes us favour that Ser2P, is, in the case of CDK9 + PP2A inhibition, one of the factors regulating mRNA CPA.

However, there are several others non-mutually exclusive factors that could also be involved in the reversal of the effect of CDK9 inhibition on mRNA CPA, including a change in pol II elongation rate, pol II processivity, and in the efficiency of co-transcriptional processes (pre-mRNA splicing and mRNA cleavage and polyadenylation). Currently, we cannot rule out any of these factors.

We hope we have now made this clear in the manuscript.

4. With the same reasoning, the model figure (Fig.8) is not very informative in my opinion.

We have updated Figure 8 and it is hopefully clearer now.

5. While the first Net-seq and 'bulk' gene analysis were done with TNFα stimulation, other experiments (ChIP-RTqPCR, phosphoproteomics, WB etc) were conducted in steady-state conditions, which is bit confusing since TNFα signalling does induce P-TEFb activity. Does TNFα increase the phosphorylation of SPT5 and/or SF3B1 or transcription of KPNB1, for example?

We used the TNFα stimulation only as a technical approach to follow what happens to the production of de novo poly(A)+ mRNA in the absence or presence of different inhibitors. As shown in Figure EV4F, we did not observe an increase in nuclear poly(A)+ mRNA for KPNB1 after TNFα stimulation, which indicates that the effect of TNFα is gene-specific.

6. Other CDKs/Cyclins, including CDK12/CycK (GD28:342, MCB35:468), and CDK11/CycL(CHM 18:560), were also reported to mediate CPA. It would be helpful to include involvement of these studies in the Discussion.

We have updated the Discussion as suggested.

7. Also, Kouzarides and colleagues demonstrated that hyperactivation of P-TEFb (by eliminating 7SKsnRNA by antisense oligos) causes global readthrough of the CPA sites (Genome Biol.14:R98). It seems that both CDK9 activation and inhibition lead to a defect in transcriptional termination. Further discussion about this would be helpful to visualise "a big picture" of CDK9-mediated transcription control.
We have updated the Discussion to include these points. Based on work of different groups (Cortazar, Sheridan et al., 2019, Parua, Booth et al., 2018, Parua, Kalan et al., 2020), hyperactivation of P-TEFb could counteract the activity of the phosphatase PP1 and cause hyperphosphorylation of SPT5, which has been shown to promote readthrough. We think that these data are in agreement with what we have found, i.e. that P-TEFb activity at the 3'end of the genes needs to be exquisitely regulated to ensure that pol II terminates in the "expected" termination region. Of note, two recent papers using a 7SK KO approach could not replicate the global readthrough effect of 7SK depletion by antisense oligos, which indicates that this effect might be transient until the cell re-establishes equilibrium (Bandiera, Wagner et al., 2021, Studniarek, Tellier et al., 2021).

Referee #2:

This study is on CDK9 functions in gene expression with a focus on RNA polyadenylation and termination. Murphy and colleagues previously suggested that CDK9 inhibition causes premature termination of pol II transcripts (Laitem eta l., 2015). This was generally confirmed and extended with respect to CDK9 functions in transcription/processing factor occupancy at 3' regions of genes. The perhaps most innovative aspect of the paper relates to possible roles of phosphatases and their interplay with CDK9. In brief, the authors propose a novel role of PP2A in 3' end formation. Along these studies also CDK9 kinase targets have been reinvestigated.

This investigation is of interest and technically state of the art. Data density makes it in some parts difficult to read though. The reader is often left alone in deciding about the relevance of data and significance of conclusions. There remain some questions about data and conclusions.
We thank the reviewer for the positive comments. We have simplified the text and hope that it is now more reader friendly.

1) One question concerns the definition of "premature termination" and "decreased polyadenylation". It is my understanding that these terms are exchangeable and the latter does not refer to downregulation of ´the process of polyadenylation. 3'READS technology measures polyadenylated 3' termini but does it in the end allow to discriminate between reduced polyadenylation activity, mitigated RNA synthesis and premature termination (Figure 1A, suppl. Figure 1A)? Please discuss and state in the ms.

We do not think these terms are interchangeable as decreased polyadenylation is not obligatorily associated with premature termination (for example, transcription readthrough occurs when co-transcriptional mRNA cleavage and polyadenylation is lost). We have defined premature termination as a loss of pol II signal closer to the poly(A) site and have shown here that in the case of CDK9 inhibition, this is coupled to a loss of (co-transcriptional) mRNA polyadenylation. This differs from more efficient termination where there is also a loss of pol II signal closer to the poly(A) site but co-transcriptional mRNA polyadenylation is unaffected or enhanced. We agree that, by itself, 3’READS does not allow discrimination between decreased polyadenylation, changes in gene expression (i.e. slower elongation rate), and premature termination of pol II before the poly(A) site is reached. However, the results of mNET-seq, ChIP, co-IP and TNFα induction followed by ChIP-qPCR, taken together, support the conclusions that CDK9 inhibition causes both premature termination of pol II and loss of polyadenylation of the

transcripts. This was particularly important to demonstrate as CDK9 inhibition could have indeed been associated with more efficient cleavage/polyadenylation which could lead in turn to premature termination.

2) For the separation of chromatin and nucleoplasm it would be helpful if the exact protocol for the actually applied method for chromatin and nucleoplasm separations was added. Attempts to find the information led through a series of publications into the last Millenium w/o clear result. This applies to protein and RNA separations (Fig 1C/D). Relatedly, I was wondering where the ribosomes go in the separation protocol (and the RNA being translated in them).

As requested, we have further described the protocol used for cellular fractionation. The ribosomes and the translated RNA should be in the cytoplasmic fraction (Neve, Burger et al., 2016).

3) Figure 1F/G: The reader is certainly somewhat lost in the complexity of data, especially the many data points in the model gene, out of which some are significant others apparently not. In my view, it would be generally important to highlight(state statistical significance for those values that are critical to the conclusions. Also, data bars are often too low to visually judge about them. It appears for example i) that the decline of pol II at +5.3 is not much different from the decline in downstream pA regions (except for pA -4.8) and ii) that CPFs drop similar to pol II at various positions in the gene. I assume that the intention is to exactly show that his is not the case by plotting ratios of CPFs to pol II. Also, the normalization procedure is unclear. How can the ratio of Xrn2/pol II be more sensitive to DRB, than Xrn2 alone at pA 4.1, if pol II shows similar sensitivity as Xrn2 at this position?

We have ratioed the CPF signal to total pol II to determine whether the CPF factor is affected more than the pol II signal as CPF factors are known to be recruited to the pol II complex (and therefore a decrease in a CPF signal might be explained by the decrease in pol II signal rather than an active loss of the CPF protein itself).

The CPSF30, Xrn2, and PAPOLA ChIP were performed on different biological replicates while pol II has been performed on every replicate. Therefore, the pol II that was initially shown in Figure 1F/G corresponded to the average pol II profile across all the replicates. We have now move in Appendix Figure S1F the unratioed CPF and their associated total pol II profile.

4) The impact of the bulky adenosine-derivative (NA) on Ser2P-Cell signaling (Fig 3A) in HEK293 appears - at least visually - as strong as in CDK9as, relative to total pol II. Also the Ser5P values seem quite comparable between the sensitive and the insensitive control lines. This is in apparent contrast to the quantitative evaluation (Fig 3B). This is

important as much of the following data is based on the CDK9as cell line. It would be helpful to have blots that support the final conclusion. Please change and/or clarify.

We have repeated some of the Ser2P and Ser5P western blots in Figure 3.

5) Figure 4B: SFB3b1 data is convincing, but can't see the effect of CDK9 blockage on SPT5-P806T. In fact, relative to total SPT5, NA seems to cause less of a decline and NA+TT is of low quality (left panel). Please clarify/exchange data!

We have added new western blots to Figure 4 for SF3B1 T142P. As SPT5 T806P was not found as a CDK9 target in our phosphoproteomics analysis, we have removed discussion of this from the manuscript and expanded the SF3B1 aspect of the manuscript.

6) Figure 7B: the authors state PP2A inhibition "fully reverses the effect of CDK9 inhibition". It is indeed intriguing that the DRB+CA levels are quite similar to the respective control values. However, how can the authors distinguish reversal at 3' ends from additive effects over the entire gene? Please discuss.

See our response to Point 3 of Reviewer 1.

Minor:

1) What are the authors referring to with "Alternatively,..." in the third sentence of the result section?

Loss of pol II signal close to the poly(A) site can be explained by either a premature termination, which is associated with a failure to produce a mature mRNA, or more efficient termination, which will be associated with the production of a mature mRNA. While the effect on pol II will look similar in both cases, i.e. a decrease/loss of pol II signal downstream of the poly(A) site, the critical difference between both modes of transcription termination is whether a mature mRNA will be produced or not.

2) It wasn't clear to me why the authors use a rather complex KPNB1 gene that apparently harbors a collection of polyA sites, with the pA at -0.4 being the prominent one, if not entirely for historical reasons? Please explain.

We are using KPNB1 as a model gene as it is ~35 kb long, which makes it ideal for investigating CDK9 function after a short inhibition, and the gene is also well expressed in our cell lines. While the *KPNB1* gene contains multiple poly(A) sites, only two sites next to each other are mostly used in our HeLa and HEK293 cells, based on the 3'READS we performed (see Figure for reviewers 1).

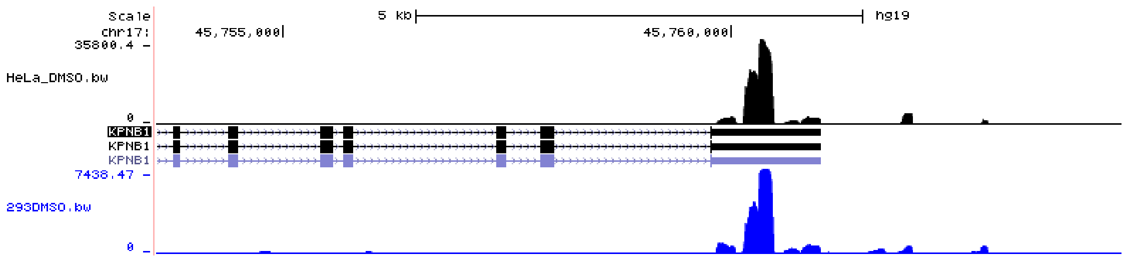

Figure for reviewers 1. Poly(A) sites usage at the 3'end of the *KPNB1* gene in HeLa cells (top, black) and HEK293 cells (bottom, blue).

3) I don't think SNS-032 is a specific for CDK9 inhibitor (p8 and supp. Fig 2c). I would thus leave the data out.

We have removed the graph as suggested.

4) Much is the based on the specificity of bulky adenosine in combination with the mutated kinase. It would be good to know whether NA has been tested on a broad panel of kinases. If so please quote.

1-NA-PP1, 1-NM-PP1, and 3MB-PP1 have been tested on a broad panel of wild-type kinase to show their specificity towards the analog-sensitive kinases (Zhang, Lopez et al., 2013), which is now cited in the manuscript. 1-NA-PP1 is not as potent or specific as 1-NM-PP1 and 3MB-PP1 but we and others failed to generate CDK9 analog-sensitive cell line with glycine replacing the phenylalanine (Gressel, Schwalb et al., 2017) and therefore made an alanine substitution together with 1-NA-PP1. Of note, after treating wild-type HEK293 cells with 1-NA-PP1, we did not detect a defect in cell growth, transcription of *KPNB1*, or on the production of poly(A)+ mRNA following TNFα induction (Figure EV2), indicating that the concentration of 1-NA-PP1 we are using does not affect transcription or polyadenylation when CDK9 is wild type.

5) The need of the proteomics data for the subsequent PP story isn't obvious to me. In fact, the authors continue with a phospho-site in SPT5 (806) that apparently wasn't even detected in their proteomics analysis. Certainly, the reader expects an explanation for the very small overlap with previous studies.

As SPT5 T806P was not identified as a CDK9 target in our phosphoproteomics analysis, we have removed discussion if this from the manuscript and expanded the SF3B1 aspect of the manuscript. We have also expanded in the Discussion on the potential reasons for the low overlap between the published CDK9 phosphoproteomics analyses and ours.

6) Figure 5: Data load is difficult to digest for the reader. Perhaps one could make them

supplemental and limit data in the text to those that make the point.
We have simplified the figures as requested.

Referee #3:

This manuscript by Tellier et al. reports novel functions of CDK9 and PP2A in 3' end processing/transcriptional termination. The work was based largely on using a set of CDK9 and PP2A inhibitors coupled with genome-wide sequencing. The key finding is that CDK9 inhibition leads to suppression of Pol II signals after the last poly(A) site and loss of CPA factors from chromatin, and these can be restored by co-inhibition of PP2A. The authors additionally showed that CDK9 can phosphorylate a group of factors that were not previously known to be its substrates, including SF3B1. The conclusions of this work are largely supported by their data. However, there are a few issues the authors need to address before the work can be accepted for publication.
We thank the reviewer for the positive comments.

1. A large number of polyA sites are located in introns. The authors should use their 3'READS data to examine their regulation.

As CDK9 inhibition promotes a loss of pol II entering productive elongation, it remains technically challenging to investigate the effect of CDK9 inhibition on intronic poly(A) site usage as only a limited number of pol II molecules will transcribe across these intronic poly(A) sites. Further analysis of the 3’READS data indicates that after CDK9 inhibition, four genes showed increased and three genes showed decreased intronic poly(A) site usage (Figure EV5).

2. For polyA sites in the last exons, the authors should examine whether there is alternative polyadenylation after CDK9 and PP2A inhibition. Based on their model, the alternative polyadenylation changes should be substantial. If so, the authors should also check if the regulation is related to pol II signal changes after the last polyA site, which is indicative of CPA defect.

From the 3’READS data, we found that, after CDK9 inhibition, there were 22 genes with increased proximal and 17 genes with increased distal poly(A) site usage (Figure EV5). To confirm the results of the 3’READS experiments, we performed qRT-PCR on three genes and confirmed the trend towards 3’UTR shortening after CDK9 inhibition. We found that PP2A inhibition has an effect on two of the three genes, with a trend towards 3’UTR lengthening, while inhibiting CDK9 and PP2A together results in a 3’UTR usage closer to the DMSO control (Figure EV5). These results indicate that CDK9 and PP2A can regulate

alternative poly(A) site usage but more work will be required to determine the mechanism behind it.

Bandiera R, Wagner RE, Britto-Borges T, Dieterich C, Dietmann S, Bornelov S, Frye M (2021) RN7SK small nuclear RNA controls bidirectional transcription of highly expressed gene pairs in skin. *Nat Commun* 12: 5864

Cortazar MA, Sheridan RM, Erickson B, Fong N, Glover-Cutter K, Brannan K, Bentley DL (2019) Control of RNA Pol II Speed by PNUTS-PP1 and Spt5 Dephosphorylation Facilitates Termination by a "Sitting Duck Torpedo" Mechanism. *Mol Cell* 76: 896-908 e4

Davidson L, Muniz L, West S (2014) 3' end formation of pre-mRNA and phosphorylation of Ser2 on the RNA polymerase II CTD are reciprocally coupled in human cells. *Genes Dev* 28: 342-56

Eifler TT, Shao W, Bartholomeeusen K, Fujinaga K, Jager S, Johnson JR, Luo Z, Krogan NJ, Peterlin BM (2015) Cyclin-dependent kinase 12 increases 3' end processing of growth factor-induced c-FOS transcripts. *Mol Cell Biol* 35: 468-78

Gressel S, Schwalb B, Decker TM, Qin W, Leonhardt H, Eick D, Cramer P (2017) CDK9-dependent RNA polymerase II pausing controls transcription initiation. *Elife* 6

Kyburz A, Friedlein A, Langen H, Keller W (2006) Direct interactions between subunits of CPSF and the U2 snRNP contribute to the coupling of pre-mRNA 3' end processing and splicing. *Mol Cell* 23: 195-205

Neve J, Burger K, Li W, Hoque M, Patel R, Tian B, Gullerova M, Furger A (2016) Subcellular RNA profiling links splicing and nuclear DICER1 to alternative cleavage and polyadenylation. *Genome Res* 26: 24-35

Parua PK, Booth GT, Sanso M, Benjamin B, Tanny JC, Lis JT, Fisher RP (2018) A Cdk9-PP1 switch regulates the elongation-termination transition of RNA polymerase II. *Nature* 558: 460-464

Parua PK, Kalan S, Benjamin B, Sanso M, Fisher RP (2020) Distinct Cdk9-phosphatase switches act at the beginning and end of elongation by RNA polymerase II. *Nat Commun* 11: 4338

Studniarek C, Tellier M, Martin PGP, Murphy S, Kiss T, Egloff S (2021) The 7SK/P-TEFb snRNP controls ultraviolet radiation-induced transcriptional reprogramming. *Cell Rep* 35: 108965

Tellier M, Zaborowska J, Caizzi L, Mohammad E, Velychko T, Schwalb B, Ferrer-Vicens I, Blears D, Nojima T, Cramer P, Murphy S (2020) CDK12 globally stimulates RNA polymerase II transcription elongation and carboxyl-terminal domain phosphorylation. *Nucleic Acids Res* 48: 7712-7727

Zhang C, Lopez MS, Dar AC, Ladow E, Finkbeiner S, Yun CH, Eck MJ, Shokat KM (2013) Structure-guided inhibitor design expands the scope of analog-sensitive kinase technology. *ACS Chem Biol* 8: 1931-8

1st Revision - Editorial Decision 8th Jul 2022

Manuscript number: EMBOR-2021-54520V2
Title: CDK9 and PP2A regulate RNA polymerase II transcription termination and coupled RNA maturation.
Author(s): Michael Tellier, Justyna Zaborowska, Jonathan Neve, Takayuki Nojima, Svenja Hester, Marjorie Fournier, Andre Furger, and Shona Murphy

Dear Drs. Murphy and Tellier,

Thank you for your patience while we have reviewed your revised manuscript. As you will see from the reports below, the referees are now all entirely positive about its publication in EMBO Reports.

We will be very pleased to publish the manuscript, pending resolution of a few minor issues/corrections have been addressed:
> We noted that a new author was added in the revision. Are the author authors aware of this and in agreement with the revised author order?
> We would ask to consider if addition of fig 1 for referees would not aid the reader. Please note that this figure will be visible within the 'review process file' of our transparent review process, u less you request it to be removed if it is to be sued in another peer reviewed publication.
> Fig 1 E: In our view reference to statistical tests are not appropriate for n=2: please display actual data with an average and no error bars.
> Fig 4A legend: please state 'biological replicates (not duplicates). In our view reference to a p value here is not appropriate.
> EV4B: In our view reference to statistical tests are not appropriate for n=2: please display actual data with an average and no error bars.
> Thank you for including details on antibodies and primers as Appendix tables and also detailed methods description. We encourage links to protocols.io (https://www.protocols.io) to allow for a more structured display of key protocols. If you have specific identifiers for any unique/new reagents reported, such as the phosphospecific AB, please add.

Our data editors had made a number of comments in the figure legends where information should be added for clarity. Please ensure these had been addressed in full.

Once you have made these minor revisions, please use the following link to submit your corrected manuscript:

Link Not Available

If all remaining corrections have been attended to, the manuscript will be published in the next available issue of EMBO reports.

Thank you for your contribution to EMBO reports.

Yours sincerely,
Bernd Pulverer
~~~~~~
Bernd Pulverer, Ph.D.
Chief Editor, EMBO Reports
EMBO
Meyerhofstrasse 1, D-69117 Heidelberg
Tel: +4962218891501
bernd.pulverer@embo.org
~~~~~~

Referee #1:

Authors adequately addresses the points I (and other reviewers) raised in the revised version, and therefore manuscript should be accepted for publication as it is. It is a very exciting study!

Referee #2:

I have no further criticism. The data look good and the presentation/discussion is much better now.

Referee #3:

The authors have addressed all my concerns.

We will be very pleased to publish the manuscript, pending resolution of a few minor issues/corrections have been addressed:
> We noted that a new author was added in the revision. Are the author authors aware of this and in agreement with the revised author order?

Yes, all the authors are aware and in agreement with the revised author order.
> We would ask to consider if addition of fig 1 for referees would not aid the reader. Please note that this figure will be visible within the 'review process file' of our transparent review process, u less you request it to be removed if it is to be sued in another peer reviewed publication.

We have now added the previous Figure 1 for referees as Appendix Figure S1F.
> Fig 1 E: In our view reference to statistical tests are not appropriate for n=2: please display actual data with an average and no error bars.

Done.
> Fig 4A legend: please state 'biological replicates (not duplicates). In our view reference to a p value here is not appropriate.

Done.
> EV4B: In our view reference to statistical tests are not appropriate for n=2: please display actual data with an average and no error bars.

Done.
> Thank you for including details on antibodies and primers as Appendix tables and also detailed methods description. We encourage links to protocols.io (https://www.protocols.io) to allow for a more structured display of key protocols. If you have specific identifiers for any unique/new reagents reported, such as the phosphospecific AB, please add.

We added protocols.io links to the key methods we used (ChIP-qPCR, mouse spike-in ChIP-seq, co-immunoprecipitation, and nuclear RNA purification).

Dr. Michael Tellier
Sir William Dunn School of Pathology
Sir William Dunn School of Pathology
University of Oxford
South Parks Road
Oxford, UK-Oxford OX1 3RE OX13RE
United Kingdom

Dear Drs. Tellier and Murphy,

Your manuscript is accepted and in production.
May I ask you to provide by email a synopsis outlining in about 5 bullet points the key findings of your paper in a manner that is complimentary to the abstract. We usually preface this with a short summary and I would suggest the following short text:
'CDK9 kinase and PP2A phosphatase regulate RNA polymerase II and the transcription the elongation complex at multiple points to couple transcriptional elongation and termination of protein-coding genes to RNA processing downstream of the early elongation checkpoint (EEC).'
We would also use this text as a summary to describe your study in the table of contents and on the journal website.

For the synopsis we also publish a graphical abstract: could you please simply fig 8 so that it is self explanatory without descriptive text in the image.

Thank you very much for publishing with EMBO Reports.

Yours sincerely,

Bernd Pulverer

~~~~~~~~~~~~~~~~~~~~~~~~~~~~~~~~
Bernd Pulverer, Ph.D.
Chief Editor, EMBO Reports
EMBO
Meyerhofstrasse 1, D-69117 Heidelberg
Tel: +4962218891501
bernd.pulverer@embo.org
~~~~~~~~~~~~~~~~~~~~~~~~~~~~~~~~

****

THINGS TO DO NOW:

You will receive proofs by e-mail approximately 2-3 weeks after all relevant files have been sent to our Production Office; you should return your corrections within 2 days of receiving the proofs.

Please inform us if there is likely to be any difficulty in reaching you at the above address at that time. Failure to meet our deadlines may result in a delay of publication, or publication without your corrections.

IMPORTANT

We will only be able to proceed with publication if you have completed and signed the correct License to Publish form and the Page Charge Authorization form (this charge is only applicable to Scientific Reports, Research Articles and Resource articles):

- PAGE CHARGE AUTHORISATION (For Scientific Reports and Articles only)
https://www.embopress.org/pb-assets/embo-site/er_apc.pdf

- LICENSE TO PUBLISH

Once your article has been received by Wiley for production the corresponding author will receive an email from Wiley's Author Services system which will ask them to log in and will present them with the appropriate license for completion.

OPEN ACCESS papers

Authors of accepted peer-reviewed original research articles may choose to pay a fee in order for their published article to be made freely accessible to all online immediately upon publication. The EMBO Open fee is fixed at $5,200 (+ VAT where applicable).

We offer two licenses for Open Access papers, CC-BY and CC-BY-NC-ND.
For more information on these licenses, please visit: https://creativecommons.org/licenses/by/3.0/ and https://creativecommons.org/licenses/by-nc-nd/3.0/deed.en_US

- PAYMENT FOR OPEN ACCESS papers

You also need to complete our payment system for Open Access articles. Please follow this link and select EMBO Reports from the drop down list and then complete the payment process:
https://authorservices.wiley.com/bauthor/onlineopen_order.asp

If you have any questions or concerns about these licenses, please feel free to contact the editorial office by replying to this email.

All further communications concerning your paper should quote reference number EMBOR-2021-54520V3 and be addressed to emboreports@wiley.com.

Should you be planning a Press Release on your article, please get in contact with emboreports@wiley.com as early as possible, in order to coordinate publication and release dates.

EMBO Press Author Checklist

Corresponding Author Name: Michael Tellier	
Journal Submitted to: EMBO Reports	
Manuscript Number: EMBOR-2021-54520	

USEFUL LINKS FOR COMPLETING THIS FORM
The EMBO Journal - Author Guidelines
EMBO Reports - Author Guidelines
Molecular Systems Biology - Author Guidelines
EMBO Molecular Medicine - Author Guidelines

Reporting Checklist for Life Science Articles (updated January 2022)
This checklist is adapted from Materials Design Analysis Reporting (MDAR) Checklist for Authors. MDAR establishes a minimum set of requirements in transparent reporting in the life sciences (see Statement of Task: 10.31222/osf.io/9sm4x). Please follow the journal's guidelines in preparing your manuscript.
Please note that a copy of this checklist will be published alongside your article.

Abridged guidelines for figures
1. Data
The data shown in figures should satisfy the following conditions:
- the data were obtained and processed according to the field's best practice and are presented to reflect the results of the experiments in an accurate and unbiased manner.
- ideally, figure panels should include only measurements that are directly comparable to each other and obtained with the same assay.
- plots include clearly labeled error bars for independent experiments and sample sizes. Unless justified, error bars should not be shown for technical replicates.
- if n<5, the individual data points from each experiment should be plotted. Any statistical test employed should be justified.
- Source Data should be included to report the data underlying figures according to the guidelines set out in the authorship guidelines on Data Presentation.

2. Captions
Each figure caption should contain the following information, for each panel where they are relevant:
- a specification of the experimental system investigated (eg cell line, species name).
- the assay(s) and method(s) used to carry out the reported observations and measurements.
- an explicit mention of the biological and chemical entity(ies) that are being measured.
- an explicit mention of the biological and chemical entity(ies) that are altered/varied/perturbed in a controlled manner.
- the exact sample size (n) for each experimental group/condition, given as a number, not a range;
- a description of the sample collection allowing the reader to understand whether the samples represent technical or biological replicates (including how many animals, litters, cultures, etc.).
- a statement of how many times the experiment shown was independently replicated in the laboratory.
- definitions of statistical methods and measures:
  - common tests, such as t-test (please specify whether paired vs. unpaired), simple $\chi 2$ tests, Wilcoxon and Mann-Whitney tests, can be unambiguously identified by name only, but more complex techniques should be described in the methods section;
  - are tests one-sided or two-sided?
  - are there adjustments for multiple comparisons?
  - exact statistical test results, e.g., P values = x but not P values < x;
  - definition of 'center values' as median or average;
  - definition of error bars as s.d. or s.e.m.

Please complete ALL of the questions below.
Select "Not Applicable" only when the requested information is not relevant for your study.

Materials

Newly Created Materials	Information included in the manuscript?	In which section is the information available? (Reagents and Tools Table, Materials and Methods, Figures, Data Availability Section)
New materials and reagents need to be available; do any restrictions apply?	Yes	Data availability Section

Antibodies	Information included in the manuscript?	In which section is the information available? (Reagents and Tools Table, Materials and Methods, Figures, Data Availability Section)
For **antibodies** provide the following information:<br>- Commercial antibodies: RRID (if possible) or supplier name, catalogue number and or/clone number<br>- Non-commercial: RRID or citation	Yes	Materials and Methods

DNA and RNA sequences	Information included in the manuscript?	In which section is the information available? (Reagents and Tools Table, Materials and Methods, Figures, Data Availability Section)
**Short novel DNA or RNA including primers, probes**: provide the sequences.	Yes	Materials and Methods

Cell materials	Information included in the manuscript?	In which section is the information available? (Reagents and Tools Table, Materials and Methods, Figures, Data Availability Section)
**Cell lines:** Provide species information, strain. Provide accession number in repository **OR** supplier name, catalog number, clone number, and/**OR** RRID.	Yes	Materials and Methods
**Primary cultures:** Provide species, strain, sex of origin, genetic modification status.	Not Applicable	
Report if the cell lines were recently **authenticated** (e.g., by STR profiling) and tested for mycoplasma contamination.	Yes	Materials and Methods

Experimental animals	Information included in the manuscript?	In which section is the information available? (Reagents and Tools Table, Materials and Methods, Figures, Data Availability Section)
**Laboratory animals or Model organisms:** Provide species, strain, sex, age, genetic modification status. Provide accession number in repository **OR** supplier name, catalog number, clone number, **OR** RRID.	Not Applicable	
**Animal observed in or captured from the field:** Provide species, sex, and age where possible.	Not Applicable	
Please detail **housing and husbandry conditions**.	Not Applicable	

Plants and microbes	Information included in the manuscript?	In which section is the information available? (Reagents and Tools Table, Materials and Methods, Figures, Data Availability Section)
**Plants:** provide species and strain, ecotype and cultivar where relevant, unique accession number if available, and source (including location for collected wild specimens).	Not Applicable	
**Microbes:** provide species and strain, unique accession number if available, and source.	Not Applicable	

Human research participants	Information included in the manuscript?	In which section is the information available? (Reagents and Tools Table, Materials and Methods, Figures, Data Availability Section)
If collected and within the bounds of privacy constraints report on age, sex and gender or ethnicity for all study participants.	Not Applicable	

Core facilities	Information included in the manuscript?	In which section is the information available? (Reagents and Tools Table, Materials and Methods, Figures, Data Availability Section)
If your work benefited from core facilities, was their service mentioned in the acknowledgments section?	Yes	Materials and Methods, Acknowledgments

Design

Study protocol	Information included in the manuscript?	In which section is the information available? (Reagents and Tools Table, Materials and Methods, Figures, Data Availability Section)
If study protocol has been **pre-registered, provide DOI in the manuscript**. For clinical trials, provide the trial registration number **OR** cite DOI.	Not Applicable	
Report the **clinical trial registration number** (at ClinicalTrials.gov or equivalent), where applicable.	Not Applicable	

Laboratory protocol	Information included in the manuscript?	In which section is the information available? (Reagents and Tools Table, Materials and Methods, Figures, Data Availability Section)
Provide DOI OR other citation details if **external detailed step-by-step protocols** are available.	Not Applicable	

Experimental study design and statistics	Information included in the manuscript?	In which section is the information available? (Reagents and Tools Table, Materials and Methods, Figures, Data Availability Section)
Include a statement about **sample size** estimate even if no statistical methods were used.	Yes	Figures
Were any steps taken to minimize the effects of subjective bias when allocating animals/samples to treatment (e.g. **randomization procedure**)? If yes, have they been described?	Not Applicable	
Include a statement about **blinding** even if no blinding was done.	Not Applicable	
Describe **inclusion/exclusion criteria** if samples or animals were excluded from the analysis. Were the criteria pre-established?<br><br>If sample or data points were omitted from analysis, report if this was due to attrition or intentional exclusion and provide justification.	Not Applicable	
For every figure, are **statistical tests** justified as appropriate? Do the data meet the assumptions of the tests (e.g., normal distribution)? Describe any methods used to assess it. Is there an estimate of variation within each group of data? Is the variance similar between the groups that are being statistically compared?	Yes	Materials and Methods and Figures

Sample definition and in-laboratory replication	Information included in the manuscript?	In which section is the information available? (Reagents and Tools Table, Materials and Methods, Figures, Data Availability Section)
In the figure legends: state number of times the experiment was **replicated** in laboratory.	Yes	Figures
In the figure legends: define whether data describe **technical or biological replicates**.	Yes	Figures

Ethics

Ethics	Information included in the manuscript?	In which section is the information available? (Reagents and Tools Table, Materials and Methods, Figures, Data Availability Section)
Studies involving **human participants**: State details of **authority granting ethics approval** (IRB or equivalent committee(s), provide reference number for approval.	Not Applicable	
Studies involving **human participants**: Include a statement confirming that **informed consent** was obtained from all subjects and that the experiments conformed to the principles set out in the WMA Declaration of Helsinki and the Department of Health and Human Services Belmont Report.	Not Applicable	
Studies involving **human participants:** For publication of **patient photos**, include a statement confirming that consent to publish was obtained.	Not Applicable	
Studies involving experimental **animals**: State details of **authority granting ethics approva**l (IRB or equivalent committee(s), provide reference number for approval. Include a statement of compliance with ethical regulations.	Not Applicable	
Studies involving **specimen and field samples:** State if relevant **permits** obtained, provide details of authority approving study; if none were required, explain why.	Not Applicable	

Dual Use Research of Concern (DURC)	Information included in the manuscript?	In which section is the information available? (Reagents and Tools Table, Materials and Methods, Figures, Data Availability Section)
Could your study fall under dual use research restrictions? Please check biosecurity documents and list of **select agents and toxins** (CDC): https://www.selectagents.gov/sat/list.htm	Not Applicable	
If you used a select agent, is the security level of the lab appropriate and reported in the manuscript?	Not Applicable	
If a study is subject to dual use research of concern regulations, is the name of the **authority granting approval and reference number** for the regulatory approval provided in the manuscript?	Not Applicable	

Reporting

The MDAR framework recommends adoption of discipline-specific guidelines, established and endorsed through community initiatives. Journals have their own policy about requiring specific guidelines and recommendations to complement MDAR.

Adherence to community standards	Information included in the manuscript?	In which section is the information available? (Reagents and Tools Table, Materials and Methods, Figures, Data Availability Section)
State if relevant guidelines or checklists (e.g., **ICMJE, MIBBI, ARRIVE, PRISMA**) have been followed or provided.	Not Applicable	
For **tumor marker prognostic studies,** we recommend that you follow the **REMARK** reporting guidelines (see link list at top right). See author guidelines, under 'Reporting Guidelines'. Please confirm you have followed these guidelines.	Not Applicable	
For **phase II and III randomized controlled trials**, please refer to the **CONSORT** flow diagram (see link list at top right) and submit the CONSORT checklist (see link list at top right) with your submission. See author guidelines, under 'Reporting Guidelines'. Please confirm you have submitted this list.	Not Applicable	

Data Availability

Data availability	Information included in the manuscript?	In which section is the information available? (Reagents and Tools Table, Materials and Methods, Figures, Data Availability Section)
Have **primary datasets** been deposited according to the journal's guidelines (see 'Data Deposition' section) and the respective accession numbers provided in the Data Availability Section?	Yes	Data Availability Section
Were **human clinical and genomic datasets** deposited in a public access-controlled repository in accordance to ethical obligations to the patients and to the applicable consent agreement?	Not Applicable	
Are **computational models** that are central and integral to a study available without restrictions in a machine-readable form? Were the relevant accession numbers or links provided?	Not Applicable	
If publicly available data were reused, provide the respective **data citations in the reference list**.	Not Applicable	
